# Clocking Injustice: Racial Disparities in Specialty Wait Times

**DOI:** 10.1111/1475-6773.14621

**Published:** 2025-04-03

**Authors:** Daniel A. Asfaw, Sarah H. Gordon, Michael Anne Kyle, Kevin N. Griffith

**Affiliations:** ^1^ Department of Health Law, Policy and Management Boston University School of Public Health Boston Massachusetts USA; ^2^ Partnered Evidence‐based Policy Research Center VA Boston Healthcare System Jamaica Plain Massachusetts USA; ^3^ Department of Health Care Policy Harvard Medical School Boston Massachusetts USA; ^4^ Department of Health Policy Vanderbilt University Medical Center Nashville Tennessee USA

**Keywords:** access to care, health equity, specialty care, veterans, wait times

## Abstract

**Objective:**

Timely access to care is associated with improved morbidity, mortality, and quality of life, making disparate access to specialty care a potential contributor to racial inequities in health outcomes. In this nationwide analysis, we quantified specialty care waits by race and ethnicity and identified potential drivers of observed disparities.

**Study Setting and Design:**

The U.S. Veterans Health Administration (VHA) provides care directly through its network of 170 medical centers and 1,193 associated outpatient clinics. In addition, the VHA pays for millions of appointments with community‐based specialists each year. We used multivariate regression models and Kitagawa‐Oaxaca‐Blinder decomposition to identify drivers of disparities between White and minoritized veterans.

**Data Sources and Analytic Sample:**

We used administrative data to identify patients referred to either VHA or community specialists during 2018–2022 for the 10 highest‐volume specialties: cardiology, dermatology, otorhinolaryngology, gastrointestinal endoscopy/gastroenterology, mental health, ophthalmology, orthopedics, podiatry, physical therapy, and urology.

**Principal Findings:**

Our sample included 6,619,517 referrals to VHA specialists and 3,753,657 referrals to community care. Black patients had the longest mean waits for VHA care (40.0 days, SD = 40.3) followed by American Indian/Alaska Native (38.6 days, SD = 40.0), Hispanic (38.4 days, SD = 38.4), Asian/Pacific Islander (38.1 days, SD = 38.0), and White patients (37.1 days, SD = 39.5).

For community care, Black patients had the longest mean waits (52.9 days, SD = 49.3) followed by Asian/Pacific Islander (46.8 days, SD 43.1), American Indian/Alaska Native (46.5 days, SD = 42.1), White (45.1 days, SD = 41.7), and Hispanic patients (42.0 days, SD = 37.6). Disparities were primarily attributable to group‐level differences in the distribution of facilities from which patients were referred and specialty mix.

**Conclusions:**

Compared to non‐Hispanic Whites, minoritized patients tended to receive referrals from facilities that had longer wait times. Policies designed to increase quality and health care supply in under‐resourced areas may be an important strategy in closing racial inequities in timely access to care.

**Summary**

- What is known on this topic
  ○ Small‐scale audit studies suggest that minoritized patients are less likely to be offered medical appointments compared to White patients.
  ○ Black patients referred to cardiology and Hispanic patients referred to orthopedics experienced longer wait times compared to White patients.
  ○ Geographic variation in health system capacity can lead to disparities in access to care, as historically marginalized patients often live in areas with fewer healthcare providers.
- What this study adds
  ○ This is the first large‐scale analysis of racial and ethnic disparities in medical care wait times across the United States.
  ○ We provide evidence of systematic differences in specialty care wait times between White and minoritized patients.
  ○ Larger racial disparities were observed for referrals to community‐based versus VHA physicians; our results suggest that the liberalization of Veterans' access to community‐based providers may have negative spillover effects on health equity.

## 1 Introduction

Inadequate access to medical care is associated with reduced health services utilization, diagnostic delays, lower health‐related quality of life, and increased mortality risk [1, 2, 3, 4]. Timely access to care is thus a core measure of health care quality and a longstanding policy priority in the United States [5]. Inequities in timely access to care between White and racial and ethnic minority communities are a potential contributor to racial disparities in health outcomes [6].

Prior research documents multiple factors contributing to racial disparities in access to care [7]. Race intersects with socioeconomic position, which can affect the generosity of insurance coverage and impose financial barriers to care [8, 9]. The supply and allocation of medical services may contribute to disparities in timely access to care since historically marginalized patients are more likely to live in geographic areas with fewer health care providers [10, 11, 12]. The time and travel required to attend an appointment are greater for minoritized patients than for White patients [13, 14]. Encouragingly, evidence from older adults newly eligible for Medicare indicates that access to universal, high‐quality insurance coverage is associated with reductions in racial and ethnic disparities in access to care and health status [15].

Access to comprehensive insurance coverage is necessary but not sufficient to close racial and ethnic health inequities [16, 17]. Within the health care system, minoritized patients frequently face discrimination from medical providers, support staff, and other racialized administrative burdens [18, 19]. Audit studies (“secret shoppers”) in select U.S. cities found that compared to White patients, patients presenting as Black are less likely to be offered an appointment for primary care or mental health services [20, 21, 22]. Evidence of differential access to care by race and ethnicity persists even within a universal coverage programs like the Veterans Health Administration (VHA). Black patients referred to cardiology and Hispanic patients referred to orthopedics experienced longer wait times compared to White patients [23, 24]. However, there are no published studies of wait time disparities for other specialties or racial and ethnic groups.

The VHA has undertaken significant efforts to improve timely access to care and offers a unique context in which to examine nonfinancial drivers of inequities. The Veterans Access, Choice and Accountability Act of 2014 and the VA MISSION Act of 2018 both allowed enrolled Veterans to seek medical care from private‐sector providers, at VHA expense, if their driving distance or wait times to see a VHA provider exceed certain thresholds [25, 26, 27]. To that end, the VHA established contracts with Optum Public Sector Solutions and TriWest Healthcare Alliance to provide care to Veterans using their existing networks of medical providers. This program is known as “community care” (CC) [28, 29]. The CC network is broad and includes nearly a half million physicians nationwide, including 57.6% of all physicians that participate in Medicare [30].

In this observational study, we examine how long Veterans wait to access ten high volume specialties, accounting for about 36.4% of all specialty care referrals. We also identify how wait times vary by Veterans' race/ethnicity and care setting (VHA or CC) and estimate how much of the observed disparities in wait times may be explained by racial and ethnic differences in observed demographic and clinical covariates (e.g., age, referral specialty, referring facility, comorbidity). This is the first analysis of racial and ethnic differences in wait times of this scale, providing the most representative snapshot of racial and ethnic disparities in wait times for medical care throughout the United States.

## 2 Methods

This study was considered exempt by the VA Boston Healthcare System Institutional Review Board and adheres to the Strengthening the Reporting of Observational Studies in Epidemiology (STROBE) reporting guideline for cross‐sectional studies. Institutional policy provides a waiver of informed consent because the research, which includes millions of Veterans, could not be practicably carried out otherwise.

### 2.1 Data Source and Population

We obtained administrative data from the VHA's Corporate Data Warehouse (CDW) to identify new patient referrals to specialty care during 2018–2022. Referrals were excluded if the patient had a previous referral from the same facility for the same specialty in the past 24 months. Thus, these referrals represent new clinical relationships, not repeat visits for established patients. We restricted our sample to the ten highest‐volume specialties in the CC program including cardiology, dermatology, otorhinolaryngology, gastroenterology, mental health, ophthalmology, orthopedics, podiatry, physical therapy, and urology. Annual data on county‐level characteristics were obtained from the Health Resources and Services Administration's Area Health Resources File.

### 2.2 Study Variables

All referrals to community‐based providers must first be approved by the local VHA medical center; we used a previously validated algorithm to identify wait times as the timestamp difference between the date of approval for the referral to specialty care and the appointment date. This approach excludes the administrative wait time of requesting approval for the referral [23, 31, 32]. We did not observe differences in approval delays by race or ethnicity for either VA or community care (Supporting Information: Appendix A1), thus these administrative burdens were excluded to facilitate fair comparisons between VHA and community care waits. Timestamps are automatically encoded by the electronic health record system versus hand‐entered, reducing the potential for errors or manipulation. Wait times greater than 365 days were restricted to limit the influence of potential outliers and hospital coding errors. Our primary independent variables of interest were patients' race and ethnicity, which were grouped into five categories: non‐Hispanic White (White), non‐Hispanic Asian American or Pacific Islander (AA/PI), non‐Hispanic Black or African American (Black), Hispanic or Latino (Hispanic), and non‐Hispanic American Indian or Alaska Native (AI/AN). All patients who identified their ethnicity as “Hispanic or Latino” were categorized as Hispanic regardless of their primary race. In more than 98% of observations, primary race is self‐reported during VHA enrollment; the remainder is entered by medical staff during episodes of care. Veterans may update their preferred race and ethnicity; annual data are provided by the VA Office of Health Equity.

We employed the Behavioral‐Ecological Framework of Healthcare Access and Navigation outlined by Ryvicker (2019) to select covariates that may plausibly be associated with appointment wait times [33]. These included *predisposing factors* such as age (grouped as < 40 to 54, 55–64, 65–79, and 80+), documented sex, and priority group (a VHA eligibility determination based primarily on household income and service‐connected disabilities) [34]. Priority groups 1 through 4 constitute those with low, moderate, or serious service‐connected disabilities; group 5 comprises those with economic hardships; group 6 includes those with various environmental exposures and special eligibility categories; and groups 7 and 8 have no service‐connected disabilities and household incomes above certain thresholds. Veterans' ages and priority groups were identified based on the date their referrals were created, using data provided by the VA Planning Systems Support Group in the CDW. We also included *need factors* that were represented via dummy variables for each of the Elixhauser comorbidities [35] and referral specialty; county‐level *neighborhood factors* including median household income, median home value, and the percentage of adults 25+ who are college graduates; and the county‐level *health care environment* including rates of specialists and family physicians per 100,000, rurality based on census tract of residence, and dummy variables to represent the VA medical centers (including their affiliated outpatient clinics) that initiated the specialty referral, collectively referred to as the *referring facilities*. We also included dummy variables for referral month and year. We used a one‐year lookback period as of January 1st of the referral year to identify comorbidities. We did not control for visit modality (in‐person vs. telehealth) which may obscure the portion of disparities rooted in differential access to broadband availability, video‐capable device ownership, and telehealth services known as the “digital divide.” [36, 37] Our unit of analysis was at the individual referral level.

### 2.3 Analytic Approach

First, to illuminate disparities in health care access, we calculated unadjusted mean wait times stratified by race and ethnicity and the setting in which care was received (VHA or CC providers). Differences between White and minoritized Veterans were assessed using two‐sided t‐tests. Second, to examine the associations between appointment wait times and individual‐level demographic or clinical characteristics, we estimated multivariate regression models using ordinary least squares stratified by race/ethnicity and care setting. Models included our full set of study covariates with standard errors clustered at the patient level.

Third, we used Kitagawa‐Oaxaca‐Blinder (KOB) decomposition to identify drivers of racial and ethnic disparities in wait times. KOB decomposition is achieved through a series of linear regressions that model the relationship between the outcome of interest (i.e., wait times) and explanatory variables (e.g., age, comorbidities) separately for each group being compared [38]. Once the models are fit, the overall differences in the outcome between groups are decomposed into two parts: the explained component, which accounts for disparities attributed to measurable individual‐level characteristics, and the unexplained component, which often reflects structural inequalities or discrimination. These models may include a large variety of potential predictors; the KOB decomposition is minimally affected by Type I error because it describes disparities using aggregate predictions, where minor spurious relationships have little impact on the overall results [39]. This decomposition has been widely applied in studies of disparities across various domains. For example, it has been used to analyze racial and gender wage gaps [40], differences in academic achievement between demographic groups [41], and highlighting how provider biases contribute to inequities in access to care or treatment outcomes [42, 43].

This decomposition estimates the extent to which disparities in wait times are attributable to group‐level differences in observed covariates (“endowment effects”), group‐level differences in the associations between covariates and wait times (“coefficient effects”), a combination of the two (“interaction effects”), or cannot be explained. This final step allows us to identify the individual contribution (in days) of each covariate to the observed disparities in wait times. More details of this procedure are available in the online eMethods. Deidentified versions of our wait time data are publicly available as part of a Mendeley Data repository [31].

## 3 Results

Our final dataset included 10,373,174 referrals to specialty care among 4,356,241 unique patients during 2018–2022. Of these, 6,619,517 referrals (3,475,169 patients) were for VHA providers and 3,753,657 (2,176,537 patients) were for CC providers (see Supporting Information: Appendix A2 for frequencies by specialty). Patients were primarily White (70.4%), male (89.4%), aged 65+ (54.7%) had 4.4 (SD: 3.1) major comorbidities, and some level of service‐connected disability (71.1%). Sample characteristics stratified by care setting (VHA vs. CC) are available in Supporting Information: Appendices A3 and A4.

### 3.1 Unadjusted Mean Waits by Race and Ethnicity

Overall, patients waited a mean of 40.9 days (SD: 41.0) for specialty care and 53.4% of referrals had appointment wait times that exceeded the VHA's access standard of 28 days (Table 1). Box plots for wait times by group are available in Supporting Information: Appendix A5. Hispanic patients had the shortest wait times overall (39.7 days, SD 38.2) followed by White (40.2 days, SD 40.5), AA/PI (41.2 days, SD 40.1), and AI/AN patients (41.8 days, SD 41.1). Black patients experienced the longest average wait times (43.6 days, SD 43.3). Mean wait times were lower for referrals to VHA (37.9 days, SD 39.7) versus community‐based (46.1, SD 42.8) specialists, both overall and for each racial/ethnic group (*p* < 0.001 for all comparisons).

**TABLE 1:** Unadjusted wait times by racial group and care setting.

Racial group	Mean (SD) wait time			% of appointment wait times over 28 days		
	Overall	VHA	CC	Overall	VHA	CC
Overall	40.9 (41.0)	37.9 (39.7)	46.1 (42.8)	53.4	49.7	59.8
Hispanic	39.7 (38.2)***	38.4 (38.4)***	42.0 (37.6)***	53.4***	51.2***	57.1***
White	40.2 (40.5)	37.1 (39.5)	45.1 (41.7)	52.6	48.5	59.1
AA/PI	41.2 (40.1)***	38.1 (38.0)***	46.8 (43.1)***	54.4***	51.0***	60.5***
AI/AN	41.8 (41.1)***	38.6 (40.0)***	46.5 (42.1)***	54.7***	50.4***	61.0***
Black	43.6 (43.3)***	40.0 (40.3)***	52.9 (49.3)***	56.0***	52.8***	64.4***

*Note*: The table presents mean wait times and shares of appointments where observed wait times exceeded 28 days, stratified by racial/ethnic group and care setting. Appointment wait times were top coded at 365 days. Differences between White and minoritized Veterans were assessed using two‐sided *t*‐tests. ***p < 0.001, **p < 0.01, *p < 0.05.

Abbreviations: CC = community care, SD = standard deviation, VHA = Veterans Health Administration.

For VHA referrals, White patients had the shortest mean wait times (37.1 days, SD 39.5) followed by AA/PI (38.1 days, SD 38.0), Hispanic (38.4 days, SD 38.4), AI/AN (38.6 days, SD 40.0), and Black patients (40.0 days, SD 40.3). Overall, 49.7% of VHA referrals exceeded the 28‐day standard; this percentage ranged from a low of 48.5% (White patients) to a high of 52.8% (Black patients).

For CC referrals, Hispanic patients had the shortest mean wait times (42.0 days, SD 37.6) followed by White (45.1 days, SD 41.7), AI/AN (46.5 days, SD 42.1), AA/PI (46.8 days, SD 43.1), and Black patients (52.9 days, SD 49.3). Overall, 59.8% of CC referrals exceeded the 28‐day standard; this percentage ranged from a low of 57.1% (Hispanic patients) to a high of 64.4% (Black patients).

### 3.2 Regression‐Adjusted Differences in Wait Times by Race and Ethnicity

In general, VHA wait times tended to be longer for patients who were male or older (Table 2). For instance, Hispanic patients aged 65–79 waited 3.9 days longer than those under age 40 (95% CI 3.5, 4.2). AA/PI patients referred to specialty care during 2022 experienced an increased wait time of 6.4 days compared to those referred during 2018 (95% CI 6.3, 6.5). There were also large differences in wait times by specialty; as an example, White patients waited 25.2 days longer for gastroenterology compared to urology (95% CI 25.1, 25.4). There were no consistent associations between wait times and priority group, referral month, or county‐level neighborhood and health care environmental characteristics (Supporting Information: Appendix A6). The associations between individual comorbidity measures tended to be small in magnitude and either significantly negative or insignificant.

**TABLE 2:** Adjusted associations between patient characteristics and wait times for VHA specialists, by racial group.

Variable	White		AA/PI		Black		Hispanic		AI/AN	
	Estimate	95% CI	Estimate	95% CI	Estimate	95% CI	Estimate	95% CI	Estimate	95% CI
Gender										
Male	Ref	Ref	Ref	Ref	Ref	Ref	Ref	Ref	Ref	Ref
Female	−0.3***	(−0.4, −0.2)	−0.4	(−1.0, 0.2)	−0.6***	(−0.8, −0.4)	−0.6***	(−1.0, −0.3)	−0.4	(−1.2, 0.4)
Rurality										
Nonrural	Ref	Ref	Ref	Ref	Ref	Ref	Ref	Ref	Ref	Ref
Rural	0.1**	(0.0, 0.2)	0.0	(−0.6, 0.7)	−0.0	(−0.2, 0.2)	0.2	(−0.2, 0.5)	−0.1	(−0.9, 0.7)
Age in years										
< 40	Ref	Ref	Ref	Ref	Ref	Ref	Ref	Ref	Ref	Ref
40–54	2.0***	(1.8, 2.1)	1.4***	(0.8, 2.0)	1.5***	(1.2, 1.8)	1.5***	(1.1, 1.8)	1.7**	(0.6, 2.7)
55–64	3.7***	(3.5, 3.8)	3.4***	(2.7, 4.1)	2.9***	(2.6, 3.1)	3.3***	(3.0, 3.7)	4.0***	(2.9, 5.0)
65–79	4.0***	(3.8, 4.1)	2.9***	(2.2, 3.6)	3.6***	(3.4, 3.9)	3.9***	(3.5, 4.2)	3.6***	(2.5, 4.7)
80+	1.4***	(1.2, 1.6)	0.9	(−0.1, 1.9)	1.0***	(0.6, 1.4)	1.0***	(0.4, 1.6)	1.8*	(0.3, 3.4)
Referral specialty										
Urology	Ref	Ref	Ref	Ref	Ref	Ref	Ref	Ref	Ref	Ref
Podiatry	−9.8***	(−10.0, −9.7)	−6.2***	(−7.1, −5.3)	−8.0***	(−8.3, −7.7)	−5.3***	(−5.8, −4.8)	−9.7***	(−11.0, −8.4)
Physical therapy	2.3***	(2.1, 2.5)	6.9***	(5.8, 7.9)	1.4***	(1.1, 1.7)	0.3	(−0.3, 0.9)	0.2	(−1.3, 1.6)
Orthopedics	−4.0***	(−4.2, −3.9)	−1.8**	(−3.0, −0.7)	−4.7***	(−5.0, −4.4)	−3.2***	(−3.8, −2.6)	−2.1**	(−3.5, −0.6)
Ophthalmology	8.4***	(8.2, 8.6)	6.9***	(5.9, 7.9)	6.6***	(6.3, 7.0)	4.4***	(3.8, 5.0)	7.6***	(6.1, 9.1)
Mental health	0.4***	(0.3, 0.5)	1.7***	(0.8, 2.7)	−0.2	(−0.5, 0.0)	1.9***	(1.4, 2.4)	0.7	(−0.6, 2.0)
GI	25.2***	(25.1, 25.4)	22.2***	(21.3, 23.1)	24.4***	(24.2, 24.7)	22.4***	(21.9, 22.9)	22.6***	(21.3, 23.8)
ENT	−3.9***	(−4.1, −3.8)	−2.5***	(−3.6, −1.4)	−5.9***	(−6.3, −5.6)	−3.7***	(−4.3, −3.1)	−5.3***	(−6.8, −3.7)
Dermatology	−3.7***	(−3.8, −3.5)	−4.4***	(−5.4, −3.4)	−6.8***	(−7.1, −6.5)	−6.5***	(−7.0, −5.9)	−6.7***	(−8.1, −5.3)
Cardiology	−2.2***	(−2.4, −2.1)	−4.6***	(−5.6, −3.5)	−5.4***	(−5.7, −5.1)	−6.0***	(−6.6, −5.4)	−4.6***	(−6.0, −3.2)
Priority group [1]										
Groups 7, 8	Ref	Ref	Ref	Ref	Ref	Ref	Ref	Ref	Ref	Ref
Group 5	−0.3***	(−0.4, −0.2)	−0.4	(−1.3, 0.6)	0.1	(−0.2, 0.3)	0.3	(−0.2, 0.8)	0.6	(−0.6, 1.8)
Groups 2, 3, 6	−0.0	(−0.1, 0.1)	0.1	(−0.7, 1.0)	0.3*	(0.0, 0.5)	0.3	(−0.2, 0.8)	−0.1	(−1.3, 1.1)
Groups 1, 4	−0.1	(−0.2, 0.0)	0.2	(−0.6, 0.9)	0.1	(−0.2, 0.3)	0.4	(−0.0, 0.8)	0.1	(−1.1, 1.2)

*Note*: The table displays regression‐adjusted associations between patient characteristics and wait times for new patient referrals to specialty care (in days), stratified by racial group. For brevity, estimates for individual comorbidities, county‐level characteristics, referral year and month, and referring facility are not shown. Differences were assessed using regression interactions with white Veterans as the reference group; see main text for more details [1]. Veterans are classified into eight priority groups as outlined by the VA. Groups 1 and 4 constitute those with serious service‐connected disabilities (greater than 50% disability or housebound); groups 2, 3, and 6 are those with low or moderate service‐connected disabilities; group 5 comprises those with economic hardships; and groups 7 and 8 have no service‐connected disabilities and household incomes above certain thresholds. Veterans cannot be members of multiple priority groups; low‐income veterans with service‐connected disabilities are categorized according to their disability rating. *p < 0.05 **p < 0.01 ***p < 0.001.

Abbreviations: AA/PI = Asian American/Pacific Islander, AI/AN = American Indian/Alaska Native, VHA = Veterans Health Administration.

CC waits tended to be shorter for patients who resided in higher‐income areas or were in priority groups 7 and 8 (no service‐connected disabilities or low‐income status) (Table 3). For instance, AI/AN patients who were in priority groups 1 or 4 (> 50% disability or housebound) waited 2.4 days longer on average than those in priority groups 7 and 8 (95% CI 1.0, 3.8). Hispanic patients from counties in the top decile for median household income waited 6.5 days less than patients in the lowest decile (95% CI −9.7, −3.4). Black patients referred during 2021 waited 13.7 days longer on average than those referred during 2018 (95% CI 13.3, 14.2). Specialty mix also played a large role; AA/PI patients referred to podiatry waited 26.9 days less than patients referred to urology (−28.5, −25.3). There were no consistent associations between wait times and sex, age, referral month, or other county‐level neighborhood and health care environmental characteristics (Supporting Information: Appendix A8). Once again, the associations between individual comorbidity measures tended to be small in magnitude and either significantly negative or insignificant.

**TABLE 3:** Adjusted associations between patient characteristics and wait times for community specialists, by racial group.

Variable	White		AA/PI		Black		Hispanic		AI/AN	
	Estimate	95% CI	Estimate	95% CI	Estimate	95% CI	Estimate	95% CI	Estimate	95% CI
Gender										
Male	Ref	Ref	Ref	Ref	Ref	Ref	Ref	Ref	Ref	Ref
Female	1.0***	(0.8, 1.2)	0.6	(−0.3, 1.5)	0.1	(−0.2, 0.5)	0.3	(−0.2, 0.8)	−0.0	(−1.2, 1.1)
Rurality										
Nonrural	Ref	Ref	Ref	Ref	Ref	Ref	Ref	Ref	Ref	Ref
Rural	0.9***	(0.8, 1.0)	1.1*	(0.2, 2.0)	0.6**	(0.2, 0.9)	1.3***	(0.9, 1.7)	2.4***	(1.4, 3.3)
Age in years										
< 40	Ref	Ref	Ref	Ref	Ref	Ref	Ref	Ref	Ref	Ref
40–54	0.5***	(0.2, 0.7)	0.6	(−0.5, 1.6)	1.2***	(0.7, 1.7)	0.5*	(0.1, 1.0)	0.3	(−1.1, 1.8)
55–64	0.2	(−0.1, 0.4)	0.3	(−0.9, 1.4)	0.8**	(0.3, 1.3)	0.6*	(0.1, 1.1)	−0.1	(−1.6, 1.3)
65–79	−0.3**	(−0.5, −0.1)	0.2	(−1.0, 1.3)	1.4***	(0.8, 1.9)	0.5	(−0.1, 1.0)	1.1	(−0.4, 2.5)
80+	−0.7***	(−1.0, −0.5)	0.7	(−0.8, 2.2)	2.2***	(1.5, 3.0)	0.8*	(0.1, 1.6)	1.3	(−0.7, 3.2)
Referral specialty										
Urology	Ref	Ref	Ref	Ref	Ref	Ref	Ref	Ref	Ref	Ref
Podiatry	−17.2***	(−17.4, −17.0)	−26.9***	(−28.4, −25.5)	−19.7***	(−20.3, −19.1)	−18.9***	(−19.6, −18.3)	−16.6***	(−18.4, −14.8)
Physical therapy	−5.0***	(−5.3, −4.7)	−16.6***	(−18.2, −14.9)	−3.6***	(−4.3, −3.0)	−9.2***	(−10.0, −8.4)	−5.2***	(−7.3, −3.1)
Orthopedics	−10.0***	(−10.3, −9.8)	−19.3***	(−20.9, −17.7)	−9.3***	(−10.0, −8.7)	−9.2***	(−9.9, −8.5)	−7.7***	(−9.6, −5.9)
Ophthalmology	−0.5***	(−0.7, −0.3)	−11.8***	(−13.2, −10.5)	0.4	(−0.1, 0.9)	−6.6***	(−7.2, −6.0)	−1.2	(−2.8, 0.5)
Mental health	−8.6***	(−8.9, −8.2)	−18.6***	(−20.4, −16.8)	−14.7***	(−15.5, −13.9)	−10.8***	(−11.6, −9.9)	−5.4***	(−7.9, −2.9)
GI	11.6***	(11.4, 11.8)	−0.6	(−2.1, 0.9)	9.3***	(8.7, 9.9)	7.1***	(6.5, 7.8)	12.6***	(10.8, 14.4)
ENT	−3.1***	(−3.4, −2.8)	−9.1***	(−10.9, −7.3)	−6.7***	(−7.5, −5.8)	−5.3***	(−6.2, −4.4)	0.0	(−2.3, 2.4)
Dermatology	5.7***	(5.5, 5.9)	−5.8***	(−7.4, −4.1)	5.0***	(4.2, 5.7)	1.8***	(1.0, 2.6)	9.1***	(7.1, 11.2)
Cardiology	−7.0***	(−7.2, −6.7)	−12.6***	(−14.3, −10.9)	−10.3***	(−11.0, −9.5)	−9.8***	(−10.5, −9.0)	−7.6***	(−9.6, −5.6)
Priority group [3]										
Groups 7, 8	Ref	Ref	Ref	Ref	Ref	Ref	Ref	Ref	Ref	Ref
Group 5	0.1	(−0.1, 0.3)	2.0**	(0.5, 3.5)	0.6*	(0.0, 1.1)	0.5	(−0.2, 1.1)	1.9*	(0.4, 3.5)
Groups 2, 3, 6	0.0	(−0.1, 0.2)	1.5*	(0.2, 2.8)	0.3	(−0.2, 0.9)	0.5	(−0.1, 1.1)	2.2**	(0.7, 3.7)
Groups 1, 4	0.2**	(0.1, 0.4)	1.5*	(0.4, 2.7)	0.0	(−0.5, 0.5)	0.3	(−0.3, 0.8)	2.4***	(1.0, 3.8)

*Note*: The table displays regression‐adjusted associations between patient characteristics and wait times for new patient referrals to specialty care (in days), stratified by racial group. For brevity, estimates for individual comorbidities, county‐level characteristics, referral year and month, and referring facility are not shown. Differences were assessed using regression interactions with white Veterans as the reference group; see main text for more details [1]. Veterans are classified into eight priority groups as outlined by the VA. Groups 1 and 4 constitute those with serious service‐connected disabilities (greater than 50% disability or housebound); groups 2, 3, and 6 are those with low or moderate service‐connected disabilities; group 5 comprises those with economic hardships; and groups 7 and 8 have no service‐connected disabilities and household incomes above certain thresholds. Veterans cannot be members of multiple priority groups; low‐income veterans with service‐connected disabilities are categorized according to their disability rating. *p < 0.05 **p < 0.01 ***p < 0.001.

Abbreviations: AA/PI = Asian American/Pacific Islander, AI/AN = American Indian/Alaska Native.

### 3.3 KOB Decomposition of Racial and Ethnic Disparities in Wait Times

**FIGURE 1:**
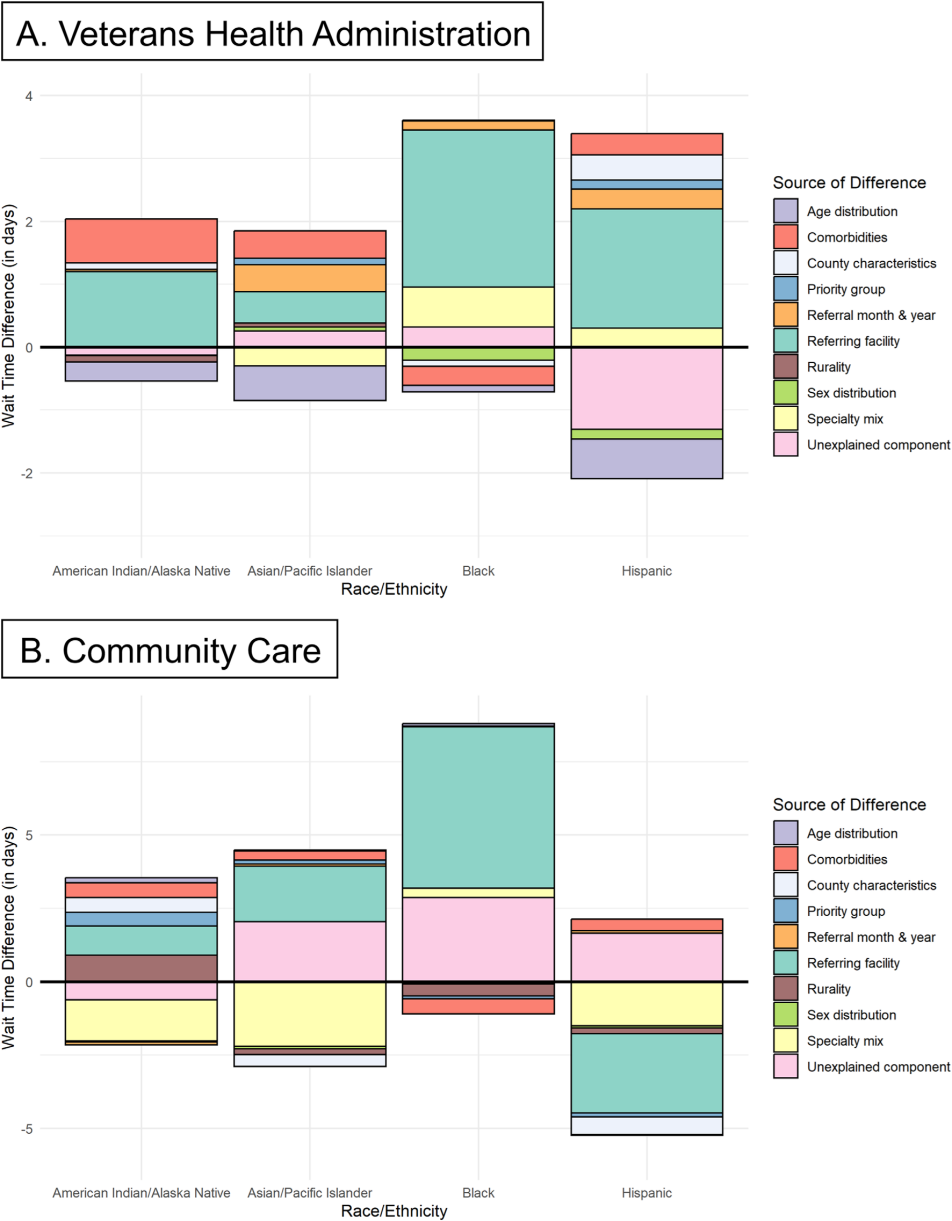
Drivers of racial disparities in appointment wait times, by care setting. *Note*: The figure combines the endowment, coefficient, and interaction effects, and displays the contributions of individual demographic and referral characteristics to the observed racial disparities in appointment wait times. White patients comprise the reference group.

Figure 1 demonstrates the drivers of disparities after combining the endowment, coefficient, and interaction effects. The detailed decomposition results suggest the largest contributors to observed wait time disparities for both VHA and community care specialists are differences in the distribution of referring facilities. Compared to White patients, Black patients waited 2.5 days longer (95% CI 1.5, 3.6) for VHA referrals and 5.5 days longer (95% CI 4.3, 6.8) for CC referrals due to group‐level differences in the distribution of referring facilities. In contrast, Hispanic patients waited 2.8 days less (95% CI 1.3, 4.3) for community care referrals for the same reason.

Lesser portions of the disparities were attributable to group‐level differences in specialty mix, comorbidity burden, and age distributions. For instance, AA/PI patients waited 2.1 days less (95% CI 1.3, 3.0) than White patients for CC appointments due to favorable differences in specialty mix. AI/AN patients waited 0.7 days less (95% CI −0.6, 1.9) than White patients for VHA specialists due to differences in comorbidity burden between the two populations. Hispanic patients waited 0.6 days less (95% CI 0.2, 0.9) than White patients for VHA specialists due to group‐level differences in age distributions.

Group‐level differences in the sex distribution, rurality, priority group, county characteristics, and referral month/year played a minimal role. Overall, our KOB decomposition results suggest that if minoritized patients had the same covariate distributions as White patients (removing the endowment effect), VHA wait times would be +0.3 days for Asian/Pacific Islander patients, +0.9 days for Black patients, −0.3 days for Hispanic patients, and +0.1 days for Native American/Alaska Native patients. Similarly, CC wait times would be +1.1 days for Asian/Pacific Islander patients, +2.5 days for Black patients, −7.4 days for Hispanic patients, and +1.0 days for Native American/Alaska Native patients. Regression output for the decomposition is provided in Supporting Information: Appendices A7 and A9.

## 4 Discussion

In this nationwide analysis of specialty care referrals, we found systematic differences in appointment wait times between White and minoritized patients. These disparities were generally minor for referrals to VHA specialists; the maximum absolute difference of 2.9 days occurred between White and Black patients, a relative difference of 7.8%. Disparities in referrals to community‐based specialists were larger; the maximum absolute difference of 10.9 days occurred between Hispanic and Black patients, a relative difference of 26.0%. The VHA's 28‐day specialty care access standard was established through an administrative decision and is not empirically based on clinical evidence; establishing a universal wait time standard for all specialties and procedures is inherently challenging. Nevertheless, we found that wait times for both VHA and CC specialists frequently exceeded this standard, especially for minoritized patients seeking CC specialty care.

While our data are limited to VHA enrollees, the VHA's community care network of 442,508 physicians includes most physicians that accept Medicare [30]. Unfortunately, health systems are not required to release data on patient wait times, and these are instead treated as trade secrets. Thus, we are unable to benchmark our findings, which represent the first comprehensive examination of disparities in wait times for specialty care in the United States.

Our regression analyses found patients' access to care varied according to sociodemographic or clinical characteristics. For instance, CC wait times were shorter in high‐ versus low‐income areas, which may reflect the tendency for doctors to settle and practice in high‐income areas [44]. Patients with a greater comorbidity burden were generally seen faster, suggesting both CC and VHA specialists prioritized access for sicker patients. Wait times were also longer for referrals that occurred after the onset of COVID‐19, comporting with results from previous work [45]. Specialty type also played a large role, with average wait time differences exceeding a month in some cases.

The largest contributing factors to racial and ethnic disparities in wait times were group‐level differences in the distribution of referring facilities. Compared to White patients, patients of color disproportionately received referrals in areas where mean wait times were longer. For instance, VHA enrollees who are Black disproportionately live in the Southeast and Mid‐Atlantic states where wait times for both VHA and community‐based specialists are longer [46, 47]. In contrast, Hispanic enrollees disproportionately live in the Southwest, where CC (but not VHA) wait times were shorter. The finding that racial and ethnic minorities are more likely to seek care from facilities with longer wait times is consistent with other evidence showing that Black patients were more likely to receive care at lower‐quality hospitals than White patients [48, 49, 50, 51]. This may reflect structural racism both in where providers choose to practice or chronic underinvestment in health care infrastructure for patients who belong to underrepresented communities.

Our study's finding that most of the disparities in both CC and VHA wait times were driven by differences in referring facility highlights the important role of local health system capacity. Place‐based policies designed to increase the quality and capacity of health care supply in under‐resourced areas may be an important strategy in reducing disparities in wait times. For example, the VHA could place greater emphasis on validated measures that identify VHA medical centers that lack capacity to meet the needs of Veterans, and direct additional staff and financial resources accordingly [52]. Further, the VA Clinical Resource Hub initiative marshals additional staffing resources towards geographic regions that are underserved for either primary, mental health, or specialty care [53, 54]. These hubs should be sufficiently resourced and promoted as a mechanism to reduce racial and ethnic disparities in appointment wait times through either telehealth or in‐person care. In addition, programs that incentivize clinicians to relocate to areas with longer wait times have the potential to improve healthcare access among racial and ethnic minority groups. The VHA also provides tens of thousands of video‐capable tablets and internet services to Veterans under its Connected Device Program, which can improve digital equity and telehealth access [55, 56].

The VHA's community care program intended to improve timely service delivery through broader access to private‐sector medical providers. In general, mean wait times were longer and racial disparities were larger in magnitude among CC versus VHA specialists. Our findings add to existing evidence that the community care program may not be achieving its intended goal of reducing Veteran wait times, and in this case, may increase race‐based disparities in access while costing the VA significantly. Future researchers could extend our work by employing a similar methodological approach to study access disparities within individual specialties, procedures, or geographic areas.

Additionally, researchers may examine what occurs within VA or CC facilities through process mapping to better understand the drivers of disparities. Investigating facilities with particularly high or low wait times could reveal whether differences are primarily due to resource allocation, operational inefficiencies, or other structural factors. Process mapping could identify specific bottlenecks or disparities in care pathways, highlighting potentially fruitful areas for targeted interventions. Furthermore, examining whether racial/ethnic groups experience different treatment and appointment wait times within specific facilities—and potential causal mechanisms including implicit bias among staff, inequitable processes, or other systemic issues—could provide actionable insights to reduce disparities and improve clinical outcomes.

## 5 Limitations

Our study design is observational; our findings should be treated as associations and may not represent causal relationships. We also are unable to identify specific facility‐level mechanisms through which race and ethnicity affect appointment wait times, or why disparities are larger for referral to CC versus VHA. While we recognize that our racial and ethnic categories are broad and these groups are not monolithic, more granular categories were not available in the VHA data. Thus, our results may mask substantial within‐group heterogeneity. Additionally, our results may not generalize to other patient populations. However, the VHA's community care network includes nearly a half million physicians, including most physicians who accept Medicare, and is broadly representative of all U.S. physicians [30]. We focused on the ten largest specialties in terms of referral volume, comprising 36.4% of all VHA and CC referrals. Our study identifies cross‐cutting disparities across ten medical specialties, and we are unable to explore each specialty in depth. However, we have provided deidentified datasets in a Mendeley Data repository so that interested researchers could conduct follow‐up analyses for individual specialties [31]. Last, while we document significant disparities in appointment wait times between racial and ethnic minorities and White patients, we cannot identify the clinical implications of treatment delays.

## 6 Conclusion

Racial and ethnic disparities in wait times for specialty care were evident within referrals to both VHA and CC clinicians. These disparities were driven primarily by attributable to group‐level differences in the distribution of facilities patients were referred from and specialty mix. For instance, minoritized Veterans received care from facilities that had longer wait times compared to White Veterans. Larger racial disparities were observed for CC referrals; our results suggest that the liberalization of Veterans' access to community‐based providers may have negative spillover effects on health equity. Further, expanding access to a limited network of CC providers may not ameliorate area‐level access barriers that contribute to racial and ethnic disparities in wait times, such as provider shortages. Place‐based interventions designed to increase the quality and capacity of health care supply in under‐resourced areas may be an important strategy in closing racial inequities in timely access to care.

## Conflicts of Interest

The authors declare no conflicts of interest.

## Supporting information


**Data S1.** Supporting Information.
