# Supplementary material for: Clocking Injustice: Racial Disparities in Specialty Wait Times
Source: Health Serv Res. 2025 Apr 3;60(5):e14621. doi: 10.1111/1475-6773.14621 (PMC12461106; doi:10.1111/1475-6773.14621)
Supplement: Supplementary file 1 — Data S1. Supporting Information. [file HESR-60-e14621-s001.docx]

**Online Appendix**

*Additional Notes on Kitigawa-Oaxaca-Blinder Decomposition*

We used Kitigawa-Oaxaca-Blinder Decomposition to break down racial and ethnic disparities in wait times into three components: endowment effects, coefficient effects, and interaction effects. Originally developed to explore discrimination in the labor market, this method has since been applied in a variety of settings including inequalities in health outcomes. The “endowment effect” measures the portion of the disparity that is attributable to group-level differences in observed covariates. To illustrate, suppose that older 1) Veterans tended to have shorter waits than younger Veterans and 2) Black Veterans tended to be younger than White Veterans on average. The portion of the Black-White disparity that is attributable to group-level differences in age distribution is an example of an endowment effect.

The “coefficient effect” measures the portion of the disparity that is attributable to differential group-level associations between the outcome (appointment wait times) and covariates. To illustrate, let’s again suppose that 1) both Black and White Veterans share the same age distribution. But now, let’s suppose that 2) older White Veterans tended to have shorter waits than younger White Veterans, and 3) this age-wait association is insignificant for other racial groups. The portion of the Black-White disparity that is attributable to group-level differences in the age-wait association is an example of a coefficient effect.

The “interaction effect” measures the portion of the disparity that is attributable to simultaneous/synergistic effects of the differential covariate levels and associations between racial groups. White Veterans served as the reference category because they had the lowest average unadjusted wait times. Some portion of the observed disparities will remain unexplained by either of the three effects.

Fourth, we unpacked the decomposition and identified the contributions of individual covariates to the observed disparities. We assessed the changes in predicted outcomes for minority groups when each covariate is set to the reference group’s (White Veterans) covariate levels (endowment effect), when each regression coefficient is set to match those found for White Veterans (coefficient effect), and the interactions between the two (interaction effect). We normalized our regressions through an averaging approach so that our estimates for categorical variables would be robust to the choice of omitted category. This final step allows us to identify the individual contribution (in days) of each covariate to the observed disparities in wait times.

| **Appendix A1. Approval wait times by racial/ethnic group and care setting** | | | | | | | | |
| --- | --- | --- | --- | --- | --- | --- | --- | --- |
|  | | **Mean (standard deviation) of wait times** | | | | | | |
| **Racial/ethnic group** | | **All Referrals** | | | **VHA** | | **CC** | |
| Overall | | 3.3 (12.6) | | | 2.1 (10.7) | | 5.3 (15.2) | |
| White | | 3.3 (12.6) | | | 2.1 (10.8) | | 5.1 (14.8) | |
| Asian/Pacific Islander | | 3.4 (12.6) *** | | | 2.2 (10.3) | | 5.7 (15.5) *** | |
| Black | | 3.2 (12.7) *** | | | 2.1 (10.5) | | 6.1 (16.8) *** | |
| Hispanic | | 3.4 (11.9) *** | | | 2.1 (9.6) *** | | 5.6 (14.7) *** | |
| Native American/Alaska Native | | 3.7 (13.3) *** | | | 2.1 (9.9) | | 5.9 (16.8) *** | |
| **Note:** VHA = Veterans Health Administration. CC = Community Care. The table presents mean (standard deviations) of approval wait times stratified by racial/ethnic group and care setting. Approval wait times were top coded at 365 days. We used paired t-tests to determine whether approval wait times differed between White and minoritized patients. ***p<0.001 **p<0.01 *p<0.05. | | | | | | | | |
| **Appendix A2. Included specialties and their relative frequencies** | | | | | | | | |
|  | **Overall** | | | **VHA** | | | **CC** | |
| **Specialty** | **Frequency** | | **Percent** | **Frequency** | | **Percent** | **Frequency** | **Percent** |
| Cardiology | 973,393 | | 9.4 | 686,855 | | 10.4 | 286,538 | 7.6 |
| Dermatology | 959,629 | | 9.3 | 650,166 | | 9.8 | 309,463 | 8.2 |
| Otorhinolaryngology | 566,198 | | 5.5 | 409,342 | | 6.2 | 156,856 | 4.2 |
| Gastroenterology | 1,748,941 | | 16.9 | 1,203,144 | | 18.2 | 545,797 | 14.5 |
| Mental Health | 858,966 | | 8.3 | 750,454 | | 11.3 | 108,512 | 2.9 |
| Ophthalmology | 1,458,268 | | 14.1 | 424,002 | | 6.4 | 1,034,266 | 27.6 |
| Orthopedics | 835,608 | | 8.1 | 474,188 | | 7.2 | 361,420 | 9.6 |
| Podiatry | 726,853 | | 7.0 | 504,299 | | 7.6 | 222,554 | 5.9 |
| Physical Therapy | 1,449,661 | | 14.0 | 967,954 | | 14.6 | 481,707 | 12.8 |
| Urology | 795,657 | | 7.8 | 549,113 | | 8.3 | 246,544 | 6.6 |
| **Total** | **10,373,174** | | **100** | **6,619,517** | | **100** | **3,753,657** | **100** |
| **Notes:** VHA = Veterans Health Administration. CC = Community Care. | | | | | | | | |

| **Appendix A3. Characteristics of referrals to Veterans Health Administration specialists** | | | | | | | |
| --- | --- | --- | --- | --- | --- | --- | --- |
| **Characteristic** | | **Overall** | **White** | **Asian/Pacific Islander** | **Black** | **Hispanic** | **American Indian/ Alaska Native** |
| Referrals | |  |  |  |  |  |  |
|  | Counts | 6619517 | 4498050 | 134762 | 1481325 | 435911 | 69469 |
|  | Proportions | 100 | 68.0 | 2.0 | 22.4 | 6.7 | 1.1 |
|  | Female (%) | 10.8 | 8.5 | 13.7 | 16.9 | 12.6 | 15.3 |
|  | Rural (%) | 30.9 | 37.8 | 16.1 | 15.4 | 15.8 | 39.9 |
| Age in years (%) | |  |  |  |  |  |  |
|  | <40 | 10.0 | 8.7 | 19.6 | 9.4 | 21.9 | 13.5 |
|  | 40-54 | 14.9 | 12.7 | 21.5 | 18.9 | 21.7 | 18.8 |
|  | 55-64 | 22.3 | 19.4 | 20.3 | 32.0 | 20.3 | 22.7 |
|  | 65-79 | 42.5 | 46.8 | 30.8 | 34.2 | 30.0 | 38.2 |
|  | 80+ | 10.3 | 12.4 | 7.8 | 5.5 | 6.2 | 6.9 |
| Priority group^1^ | |  |  |  |  |  |  |
|  | % in groups 1, 4 | 50.1 | 46.3 | 64.1 | 57.1 | 60.0 | 56.2 |
|  | % in groups 2, 3, 6 | 21.0 | 22.5 | 17.9 | 17.6 | 17.8 | 18.6 |
|  | % in group 5 | 17.8 | 18.8 | 10.3 | 16.6 | 14.0 | 17.4 |
|  | % in groups 7, 8 | 11.2 | 12.4 | 7.7 | 8.7 | 8.1 | 7.8 |
| Comorbid Conditions (%) | |  |  |  |  |  |  |
|  | AIDS/HIV | 0.8 | 0.4 | 0.4 | 1.7 | 0.8 | 0.6 |
|  | Alcohol abuse | 13.4 | 12.3 | 12.3 | 16.6 | 15.0 | 15.7 |
|  | Anemia deficiency | 16.2 | 15.3 | 15.3 | 20.1 | 13.2 | 14.8 |
|  | Autoimmune conditions | 4.7 | 4.9 | 4.9 | 4.4 | 4.8 | 5.1 |
|  | Blood loss anemia | 1.3 | 1.2 | 1.2 | 1.6 | 1.1 | 1.3 |
|  | Leukemia | 0.7 | 0.8 | 0.8 | 0.4 | 0.4 | 0.6 |
|  | Lymphoma | 1.1 | 1.1 | 1.1 | 1.0 | 0.8 | 0.8 |
|  | Metastatic Cancer | 1.3 | 1.4 | 1.4 | 1.2 | 0.8 | 1.1 |
|  | Solid tumor without metastasis, in situ | 5.5 | 5.9 | 5.9 | 5.1 | 3.1 | 4.3 |
|  | Solid tumor without metastasis, malignant | 11.3 | 11.9 | 11.9 | 11.1 | 6.8 | 8.9 |
|  | Cerebrovascular disease, present on admission | 26.3 | 28.9 | 28.9 | 21.9 | 17.2 | 23.0 |
|  | Cerebrovascular disease, sequela | 2.4 | 2.3 | 2.3 | 3.1 | 1.9 | 2.2 |
|  | Coagulopathy | 3.8 | 4.1 | 4.1 | 3.1 | 3.2 | 3.5 |
|  | Dementia | 3.6 | 3.9 | 3.9 | 3.2 | 2.8 | 3.1 |
|  | Depression | 40.6 | 37.9 | 37.9 | 45.8 | 49.3 | 46.6 |
|  | Diabetes with chronic complications | 24.1 | 23.7 | 23.7 | 25.8 | 23.0 | 24.9 |
|  | Diabetes without chronic complications | 32.0 | 31.2 | 31.2 | 34.8 | 30.8 | 32.4 |
|  | Drug abuse | 8.8 | 7.3 | 7.3 | 13.5 | 8.5 | 10.2 |
|  | Fluid and electrolyte disorders | 11.2 | 11.1 | 11.1 | 12.5 | 9.0 | 11.2 |
|  | Heart Failure | 10.4 | 10.9 | 10.9 | 10.4 | 6.2 | 9.1 |
|  | Homelessness | 4.7 | 3.4 | 3.4 | 8.7 | 4.8 | 7.1 |
|  | Hypertension with chronic complications | 13.6 | 13.1 | 13.1 | 16.8 | 9.6 | 11.1 |
|  | Hypertension without chronic complications | 65.7 | 65.8 | 65.8 | 70.2 | 52.4 | 60.1 |
|  | Liver disease, mild | 10.1 | 9.7 | 9.7 | 11.0 | 12.1 | 10.4 |
|  | Liver disease, severe | 1.0 | 1.1 | 1.1 | 0.7 | 1.3 | 1.3 |
|  | Liver disease, unknown severity | 0.1 | 0.1 | 0.1 | 0.1 | 0.1 | 0.1 |
|  | Chronic pulmonary disease | 22.3 | 24.3 | 24.3 | 18.9 | 14.8 | 22.6 |
|  | Neurological disorders affecting movement | 4.1 | 5.1 | 5.1 | 1.9 | 2.9 | 4.0 |
|  | Other neurological disorders | 6.6 | 6.9 | 6.9 | 5.8 | 5.7 | 6.6 |
|  | Seizures and epilepsy | 2.6 | 2.6 | 2.6 | 2.9 | 2.3 | 3.1 |
|  | Obesity | 30.1 | 29.5 | 29.5 | 31.3 | 33.3 | 31.5 |
|  | Paralysis | 2.1 | 1.9 | 1.9 | 2.8 | 1.6 | 1.9 |
|  | Peripheral vascular disease | 14.5 | 16.4 | 16.4 | 10.9 | 8.7 | 12.0 |
|  | Psychoses | 13.2 | 12.7 | 12.7 | 14.4 | 13.9 | 14.9 |
|  | Pulmonary circulation disorder | 2.8 | 2.9 | 2.9 | 3.1 | 1.7 | 2.5 |
|  | Renal failure, moderate | 10.7 | 10.7 | 10.7 | 12.1 | 7.1 | 8.5 |
|  | Renal failure, severe | 2.5 | 2.1 | 2.1 | 3.8 | 2.3 | 2.5 |
|  | Renal failure, unknown severity | 2.7 | 2.3 | 2.3 | 4.0 | 2.0 | 2.1 |
|  | Hypothyroidism | 10.1 | 12.0 | 12.0 | 5.1 | 8.6 | 10.5 |
|  | Other thyroid disorders | 3.3 | 3.1 | 3.1 | 4.1 | 2.4 | 3.1 |
|  | Peptic ulcer disease | 1.3 | 1.3 | 1.3 | 1.3 | 1.1 | 1.3 |
|  | Valvular disease | 5.5 | 6.4 | 6.4 | 3.9 | 3.4 | 4.6 |
|  | Weight loss | 4.8 | 4.7 | 4.7 | 5.7 | 3.4 | 4.4 |
| **Note:** Categorizations represent Veterans' self-reported primary race and ethnicity. All Hispanic Veterans were counted as Hispanic, regardless of primary race. ^1^Patients were classified into eight priority groups as outlined by the VA. Groups 1 and 4 constitute those with serious service-connected disabilities (greater than 50% disability or housebound); groups 2, 3, and 6 are those with low or moderate service-connected disabilities; group 5 comprises those with economic hardships; and groups 7 and 8 have no service-connected disabilities and household incomes above certain thresholds. | | | | | | | |

| **Appendix A4. Characteristics of referrals to community-based specialists** | | | | | | | |
| --- | --- | --- | --- | --- | --- | --- | --- |
| **Characteristic** | | **Overall** | **White** | **Asian/Pacific Islander** | **Black** | **Hispanic** | **American Indian/ Alaska Native** |
| Referrals | |  |  |  |  |  |  |
|  | Counts | 3,753,657 | 2,803,575 | 75,338 | 564,836 | 261,239 | 48,669 |
|  | Proportions | 100 | 74.7 | 2.0 | 15.1 | 7.0 | 1.3 |
|  | Female (%) | 10.2 | 8.2 | 13.2 | 18.6 | 11.5 | 14.2 |
|  | Rurality (%) | 48.0 | 54.7 | 30.0 | 27.1 | 24.7 | 56.7 |
| Age in years (%) | |  |  |  |  |  |  |
|  | <40 | 7.6 | 6.4 | 13.4 | 8.3 | 16.9 | 10.1 |
|  | 40-54 | 13.7 | 11.4 | 20.0 | 20.5 | 21.6 | 17.6 |
|  | 55-64 | 20.8 | 18.4 | 21.0 | 33.0 | 20.3 | 21.5 |
|  | 65-79 | 45.7 | 49.6 | 36.4 | 33.4 | 34.2 | 42.6 |
|  | 80+ | 12.1 | 14.2 | 9.2 | 4.8 | 7.0 | 8.2 |
| Priority group^1^ | |  |  |  |  |  |  |
|  | % in groups 1, 4 | 51.3 | 47.5 | 66.8 | 62.5 | 62.0 | 57.1 |
|  | % in groups 2, 3, 6 | 21.1 | 22.4 | 17.5 | 16.4 | 18.5 | 18.4 |
|  | % in group 5 | 17.0 | 18.2 | 9.1 | 14.1 | 12.8 | 17.0 |
|  | % in groups 7, 8 | 10.6 | 11.9 | 6.6 | 7.0 | 6.7 | 7.4 |
| Comorbid Conditions (%) | |  |  |  |  |  |  |
|  | AIDS/HIV | 0.4 | 0.3 | 0.3 | 1.2 | 0.5 | 0.3 |
|  | Alcohol abuse | 11.0 | 10.2 | 10.2 | 13.9 | 13.6 | 14.3 |
|  | Anemia deficiency | 14.7 | 14.0 | 14.0 | 18.9 | 13.3 | 13.4 |
|  | Autoimmune conditions | 4.5 | 4.5 | 4.5 | 4.3 | 4.7 | 4.8 |
|  | Blood loss anemia | 1.0 | 0.9 | 0.9 | 1.3 | 1.0 | 1.0 |
|  | Leukemia | 0.7 | 0.8 | 0.8 | 0.4 | 0.4 | 0.6 |
|  | Lymphoma | 1.0 | 1.0 | 1.0 | 0.9 | 0.6 | 0.7 |
|  | Metastatic Cancer | 1.0 | 1.1 | 1.1 | 0.9 | 0.6 | 0.8 |
|  | Solid tumor without metastasis, in situ | 5.8 | 6.3 | 6.3 | 4.9 | 3.2 | 4.5 |
|  | Solid tumor without metastasis, malignant | 10.7 | 11.3 | 11.3 | 10.0 | 6.4 | 8.1 |
|  | Cerebrovascular disease, present on admission | 25.0 | 27.3 | 27.3 | 18.6 | 15.9 | 21.3 |
|  | Cerebrovascular disease, sequela | 2.0 | 2.0 | 2.0 | 2.4 | 1.7 | 1.9 |
|  | Coagulopathy | 3.3 | 3.5 | 3.5 | 2.5 | 3.2 | 2.7 |
|  | Dementia | 3.2 | 3.4 | 3.4 | 2.5 | 2.7 | 2.8 |
|  | Depression | 36.2 | 33.6 | 33.6 | 44.1 | 44.7 | 41.2 |
|  | Diabetes with chronic complications | 24.6 | 24.1 | 24.1 | 26.1 | 26.4 | 25.7 |
|  | Diabetes without chronic complications | 32.9 | 32.2 | 32.2 | 35.4 | 34.6 | 34.1 |
|  | Drug abuse | 6.7 | 5.9 | 5.9 | 10.9 | 6.8 | 8.1 |
|  | Fluid and electrolyte disorders | 9.0 | 9.0 | 9.0 | 10.0 | 7.5 | 9.0 |
|  | Heart Failure | 9.5 | 10.1 | 10.1 | 8.6 | 5.8 | 8.6 |
|  | Homelessness | 3.1 | 2.3 | 2.3 | 6.5 | 3.3 | 5.7 |
|  | Hypertension with chronic complications | 11.9 | 11.7 | 11.7 | 14.0 | 9.6 | 10.0 |
|  | Hypertension without chronic complications | 67.1 | 67.5 | 67.5 | 71.1 | 57.1 | 61.9 |
|  | Liver disease, mild | 8.0 | 7.7 | 7.7 | 8.5 | 10.3 | 8.5 |
|  | Liver disease, severe | 0.8 | 0.9 | 0.9 | 0.6 | 1.0 | 0.9 |
|  | Liver disease, unknown severity | 0.1 | 0.1 | 0.1 | 0.1 | 0.1 | 0.1 |
|  | Chronic pulmonary disease | 22.6 | 24.4 | 24.4 | 17.9 | 14.3 | 22.4 |
|  | Neurological disorders affecting movement | 4.2 | 4.9 | 4.9 | 1.8 | 2.8 | 3.6 |
|  | Other neurological disorders | 5.4 | 5.6 | 5.6 | 4.6 | 4.6 | 5.3 |
|  | Seizures and epilepsy | 2.3 | 2.3 | 2.3 | 2.5 | 2.1 | 2.8 |
|  | Obesity | 28.6 | 27.7 | 27.7 | 31.9 | 31.9 | 30.0 |
|  | Paralysis | 1.6 | 1.5 | 1.5 | 2.2 | 1.3 | 1.6 |
|  | Peripheral vascular disease | 13.3 | 14.7 | 14.7 | 9.3 | 8.4 | 10.9 |
|  | Psychoses | 11.3 | 10.8 | 10.8 | 13.4 | 11.5 | 12.9 |
|  | Pulmonary circulation disorder | 2.4 | 2.5 | 2.5 | 2.4 | 1.4 | 2.0 |
|  | Renal failure, moderate | 10.4 | 10.7 | 10.7 | 10.8 | 7.9 | 8.3 |
|  | Renal failure, severe | 2.2 | 2.0 | 2.0 | 3.2 | 2.2 | 2.3 |
|  | Renal failure, unknown severity | 2.2 | 2.0 | 2.0 | 3.2 | 1.9 | 1.7 |
|  | Hypothyroidism | 11.4 | 12.7 | 12.7 | 5.6 | 10.1 | 10.9 |
|  | Other thyroid disorders | 2.8 | 2.7 | 2.7 | 3.7 | 2.0 | 2.7 |
|  | Peptic ulcer disease | 1.0 | 1.1 | 1.1 | 1.0 | 0.9 | 1.0 |
|  | Valvular disease | 5.0 | 5.7 | 5.7 | 3.2 | 3.0 | 4.1 |
|  | Weight loss | 4.0 | 4.0 | 4.0 | 4.6 | 2.8 | 3.8 |
| **Note:** Categorizations represent Veterans' self-reported primary race and ethnicity. All Hispanic Veterans were counted as Hispanic, regardless of primary race. ^1^Patients were classified into eight priority groups as outlined by the VA. Groups 1 and 4 constitute those with serious service-connected disabilities (greater than 50% disability or housebound); groups 2, 3, and 6 are those with low or moderate service-connected disabilities; group 5 comprises those with economic hardships; and groups 7 and 8 have no service-connected disabilities and household incomes above certain thresholds. | | | | | | | |

**Appendix A5. Box plots of wait times, by setting and racial group.**

*VA Referrals*


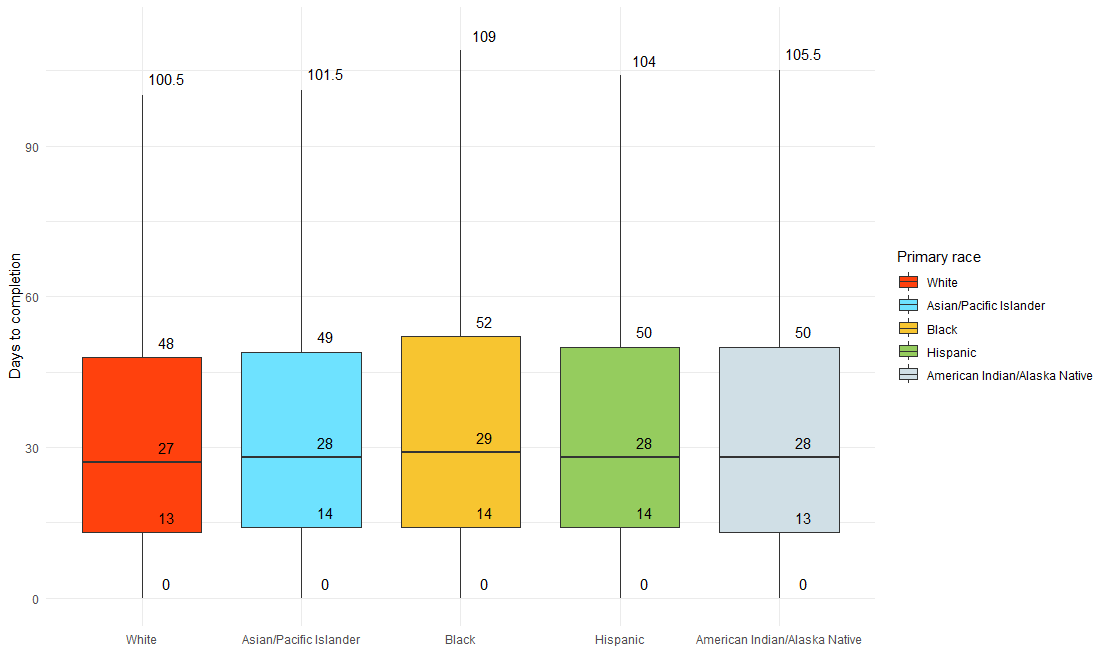


*Community Care Referrals*


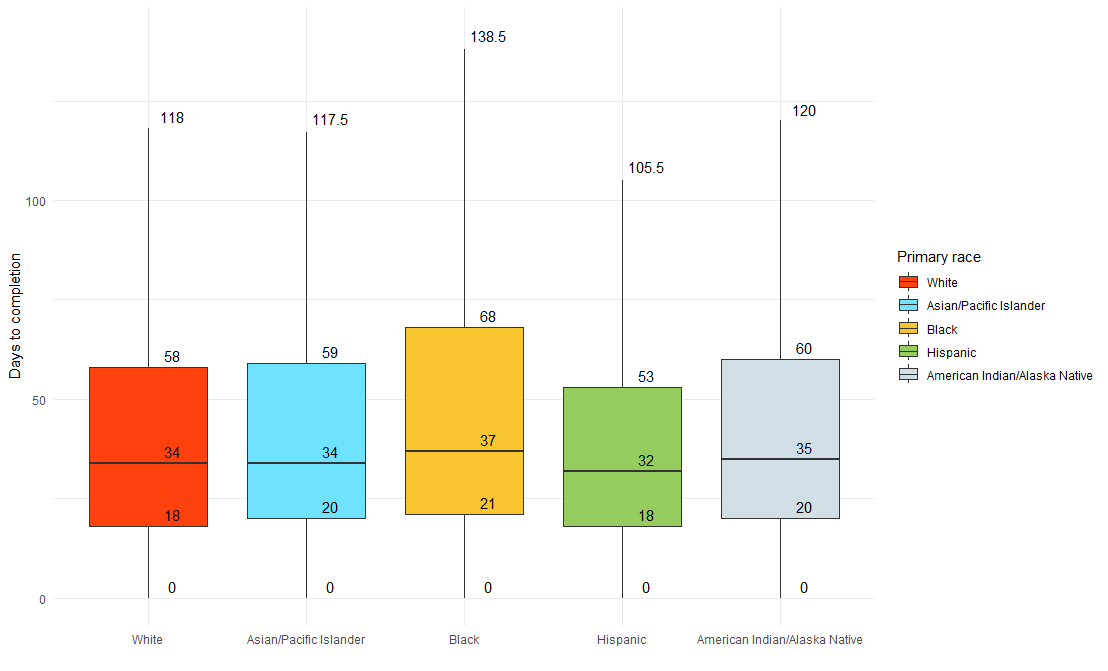


| **Appendix A6. Adjusted associations between Veteran characteristics and wait times for appointments with VHA specialists, by racial group** | | | | | | | | | | | |
| --- | --- | --- | --- | --- | --- | --- | --- | --- | --- | --- | --- |
| **Characteristic** | | **White** | | **AA/PI** | | **Black** | | **Hispanic** | | **AI/AN** | |
|  |  | **Estimate** | **95% CI** | **Estimate** | **95% CI** | **Estimate** | **95% CI** | **Estimate** | **95% CI** | **Estimate** | **95% CI** |
| Gender | |  |  |  |  |  |  |  |  |  |  |
|  | Male | ref | ref | ref | ref | ref | ref | ref | ref | ref | ref |
|  | Female | -0.3*** | (-0.4, -0.1) | -0.1 | (-0.7, 0.5) | -0.3** | (-0.6, -0.1) | -0.4* | (-0.7, -0.0) | -0.1 | (-1.0, 0.7) |
| Rurality | |  |  |  |  |  |  |  |  |  |  |
|  | Non-Rural | ref | ref | ref | ref | ref | ref | ref | ref | ref | ref |
|  | Rural | 0.1** | (0.0, 0.2) | -0.1 | (-0.8, 0.6) | -0.1 | (-0.4, 0.1) | 0.0 | (-0.3, 0.4) | -0.2 | (-1.0, 0.5) |
| Age in years | |  |  |  |  |  |  |  |  |  |  |
|  | <40 | ref | ref | ref | ref | ref | ref | ref | ref | ref | ref |
|  | 40-54 | 2.0*** | (1.8, 2.1) | -0.6 | (-1.2, 0.0) | -0.5*** | (-0.7, -0.2) | -0.5** | (-0.8, -0.2) | -0.3 | (-1.2, 0.6) |
|  | 55-64 | 3.7*** | (3.5, 3.8) | -0.3 | (-1.0, 0.4) | -0.8*** | (-1.1, -0.5) | -0.3 | (-0.7, 0.1) | 0.3 | (-0.7, 1.3) |
|  | 65-79 | 4.0*** | (3.8, 4.1) | -1.1** | (-1.8, -0.4) | -0.3* | (-0.6, -0.0) | -0.1 | (-0.5, 0.3) | -0.4 | (-1.4, 0.7) |
|  | 80+ | 1.4*** | (1.2, 1.6) | -0.5 | (-1.5, 0.5) | -0.4* | (-0.8, -0.0) | -0.4 | (-1.0, 0.2) | 0.5 | (-1.1, 2.0) |
| Priority Group^1^ | |  |  |  |  |  |  |  |  |  |  |
|  | Groups 1, 4 | -0.1 | (-0.2, 0.0) | 0.3 | (-0.5, 1.0) | 0.1 | (-0.1, 0.4) | 0.5* | (0.1, 1.0) | 0.2 | (-1.0, 1.3) |
|  | Groups 2, 3, 6 | -0.0 | (-0.1, 0.1) | 0.1 | (-0.7, 1.0) | 0.3* | (0.0, 0.6) | 0.3 | (-0.2, 0.8) | -0.1 | (-1.4, 1.1) |
|  | Group 5 | -0.3*** | (-0.4, -0.2) | -0.1 | (-1.0, 0.9) | 0.3* | (0.0, 0.6) | 0.6* | (0.1, 1.1) | 0.9 | (-0.4, 2.2) |
|  | Groups 7, 8 | ref | ref | ref | ref | ref | ref | ref | ref | ref | ref |
| Specialty | |  |  |  |  |  |  |  |  |  |  |
|  | Cardiology | -2.2*** | (-2.4, -2.1) | -2.4*** | (-3.3, -1.4) | -3.2*** | (-3.5, -2.9) | -3.8*** | (-4.3, -3.3) | -2.4*** | (-3.7, -1.1) |
|  | Dermatology | -3.7*** | (-3.8, -3.5) | -0.8 | (-1.6, 0.1) | -3.1*** | (-3.4, -2.8) | -2.8*** | (-3.3, -2.3) | -3.0*** | (-4.3, -1.8) |
|  | Otorhinolaryngology | -3.9*** | (-4.1, -3.7) | 1.4** | (0.4, 2.4) | -2.0*** | (-2.3, -1.7) | 0.2 | (-0.3, 0.7) | -1.4* | (-2.6, -0.1) |
|  | Gastroenterology | 25.2*** | (25.1, 25.4) | -3.0*** | (-4.0, -2.1) | -0.8*** | (-1.1, -0.5) | -2.9*** | (-3.4, -2.3) | -2.7*** | (-4.0, -1.3) |
|  | Mental health | 0.4*** | (0.2, 0.6) | 1.3** | (0.5, 2.2) | -0.7*** | (-0.9, -0.4) | 1.5*** | (1.0, 2.0) | 0.3 | (-0.9, 1.5) |
|  | Ophthalmology | 8.4*** | (8.2, 8.6) | -1.5* | (-2.6, -0.4) | -1.7*** | (-2.2, -1.3) | -4.0*** | (-4.7, -3.4) | -0.8 | (-2.5, 0.8) |
|  | Orthopedics | -4.0*** | (-4.2, -3.9) | 2.2*** | (1.3, 3.2) | -0.7*** | (-1.0, -0.4) | 0.8** | (0.3, 1.3) | 2.0** | (0.7, 3.2) |
|  | Physical therapy | 2.3*** | (2.1, 2.5) | 4.6*** | (3.5, 5.6) | -0.9*** | (-1.2, -0.6) | -2.0*** | (-2.5, -1.4) | -2.1** | (-3.5, -0.8) |
|  | Podiatry | -9.8*** | (-10.0, -9.7) | 3.6*** | (2.9, 4.4) | 1.8*** | (1.6, 2.0) | 4.5*** | (4.1, 4.9) | 0.1 | (-0.9, 1.1) |
|  | Urology | ref | ref | ref | ref | ref | ref | ref | ref | ref | ref |
| Comorbidities | |  |  |  |  |  |  |  |  |  |  |
|  | AIDS/HIV | 0.5 | (-0.0, 1.0) | -2.0 | (-4.7, 0.6) | -1.2*** | (-2.0, -0.5) | -0.5 | (-1.9, 0.8) | -0.9 | (-4.8, 3.1) |
|  | Alcohol abuse | -0.5*** | (-0.7, -0.4) | 0.2 | (-0.5, 0.9) | 0.1 | (-0.1, 0.4) | 0.1 | (-0.3, 0.4) | -0.3 | (-1.2, 0.6) |
|  | Anemia deficiency | -1.2*** | (-1.3, -1.1) | 1.0** | (0.3, 1.7) | 0.3** | (0.1, 0.5) | 0.2 | (-0.2, 0.5) | 0.3 | (-0.6, 1.2) |
|  | Autoimmune conditions | 0.0 | (-0.1, 0.2) | -1.0* | (-2.0, -0.1) | -0.2 | (-0.5, 0.2) | 0.0 | (-0.5, 0.5) | 0.7 | (-0.6, 2.1) |
|  | Blood loss anemia | -2.4*** | (-2.8, -2.1) | 1.6 | (-0.8, 3.9) | 0.4 | (-0.2, 1.0) | 0.0 | (-1.1, 1.2) | 1.0 | (-2.0, 4.0) |
|  | Leukemia | -0.9*** | (-1.3, -0.5) | -2.0 | (-4.6, 0.7) | -0.2 | (-1.3, 0.8) | -1.4 | (-3.2, 0.4) | -1.0 | (-4.8, 2.8) |
|  | Lymphoma | -1.7*** | (-2.0, -1.3) | 1.1 | (-1.2, 3.3) | 0.3 | (-0.4, 1.0) | -0.6 | (-1.9, 0.7) | 1.5 | (-1.8, 4.7) |
|  | Metastatic Cancer | -3.9*** | (-4.2, -3.5) | 0.8 | (-1.4, 3.1) | 0.8* | (0.1, 1.5) | 1.1 | (-0.3, 2.4) | -0.6 | (-3.5, 2.3) |
|  | Solid tumor without metastasis, in situ | 0.2* | (0.0, 0.3) | -0.7 | (-1.8, 0.5) | -0.1 | (-0.5, 0.2) | 0.3 | (-0.5, 1.0) | -0.7 | (-2.2, 0.9) |
|  | Solid tumor without metastasis, malignant | -0.6*** | (-0.7, -0.5) | 0.2 | (-0.7, 1.1) | -0.0 | (-0.3, 0.2) | -0.8** | (-1.3, -0.2) | 1.2 | (-0.0, 2.4) |
|  | Cerebrovascular disease, present on admission | -0.7*** | (-0.8, -0.6) | 0.2 | (-0.3, 0.8) | -0.2 | (-0.4, 0.0) | -0.0 | (-0.3, 0.3) | 0.0 | (-0.7, 0.8) |
|  | Cerebrovascular disease, sequela | -0.1 | (-0.3, 0.2) | -1.2 | (-2.8, 0.4) | 0.2 | (-0.4, 0.7) | 0.1 | (-0.9, 1.0) | -0.2 | (-2.4, 2.0) |
|  | Coagulopathy | -0.8*** | (-0.9, -0.6) | -0.6 | (-1.9, 0.6) | 0.3 | (-0.2, 0.7) | 0.4 | (-0.3, 1.1) | -0.5 | (-2.2, 1.1) |
|  | Dementia | -0.1 | (-0.3, 0.1) | -1.4* | (-2.6, -0.1) | -0.4 | (-0.8, 0.1) | -0.8* | (-1.5, -0.1) | 1.8 | (-0.2, 3.7) |
|  | Depression | -0.4*** | (-0.4, -0.3) | 0.4 | (-0.1, 0.8) | 0.1 | (-0.0, 0.3) | 0.4** | (0.1, 0.6) | 0.3 | (-0.3, 1.0) |
|  | Diabetes with chronic complications | 0.0 | (-0.1, 0.2) | 0.7* | (0.0, 1.4) | -0.2 | (-0.4, 0.0) | 0.2 | (-0.2, 0.6) | -0.9 | (-1.8, 0.1) |
|  | Diabetes without chronic complications | 0.8*** | (0.7, 0.9) | -0.4 | (-1.0, 0.2) | 0.1 | (-0.1, 0.3) | -0.4* | (-0.8, -0.1) | 0.8 | (-0.1, 1.6) |
|  | Drug abuse | -1.0*** | (-1.1, -0.8) | -1.8*** | (-2.7, -0.9) | 0.2 | (-0.1, 0.5) | -0.1 | (-0.6, 0.3) | 0.7 | (-0.4, 1.7) |
|  | Fluid and electrolyte disorders | -1.6*** | (-1.7, -1.5) | 0.3 | (-0.5, 1.1) | 0.2 | (-0.1, 0.4) | 0.1 | (-0.4, 0.5) | -0.8 | (-1.8, 0.2) |
|  | Heart Failure | 0.4*** | (0.3, 0.5) | -0.8 | (-1.7, 0.1) | 0.2 | (-0.1, 0.4) | -0.7* | (-1.3, -0.1) | -0.7 | (-1.9, 0.5) |
|  | Homelessness | -2.2*** | (-2.4, -2.0) | 0.5 | (-0.6, 1.5) | 0.1 | (-0.2, 0.4) | 0.4 | (-0.2, 0.9) | -0.4 | (-1.5, 0.7) |
|  | Hypertension with chronic complications | -0.2*** | (-0.3, -0.1) | -0.0 | (-0.9, 0.8) | -0.2 | (-0.4, 0.1) | 0.2 | (-0.3, 0.7) | -0.0 | (-1.2, 1.1) |
|  | Hypertension without chronic complications | 0.3*** | (0.2, 0.4) | -0.1 | (-0.6, 0.4) | 0.0 | (-0.2, 0.2) | 0.2 | (-0.1, 0.5) | 0.9* | (0.2, 1.6) |
|  | Liver disease, mild | -1.1*** | (-1.3, -1.0) | -0.4 | (-1.1, 0.3) | 0.0 | (-0.2, 0.3) | 0.2 | (-0.2, 0.6) | -0.1 | (-1.2, 0.9) |
|  | Liver disease, severe | 0.2 | (-0.1, 0.6) | -1.4 | (-4.1, 1.4) | -0.3 | (-1.2, 0.6) | -0.7 | (-2.0, 0.6) | 2.9 | (-0.3, 6.1) |
|  | Liver disease, unknown severity | -1.9*** | (-3.0, -0.8) | 2.4 | (-4.0, 8.8) | -1.6 | (-3.7, 0.5) | -0.4 | (-3.9, 3.0) | -2.8 | (-10.6, 4.9) |
|  | Chronic pulmonary disease | -0.2*** | (-0.3, -0.1) | 0.6* | (0.1, 1.2) | 0.0 | (-0.2, 0.2) | 0.2 | (-0.2, 0.5) | 0.5 | (-0.3, 1.2) |
|  | Neurological disorders affecting movement | -0.3*** | (-0.5, -0.2) | -0.1 | (-1.2, 1.1) | -0.0 | (-0.5, 0.4) | -0.3 | (-1.0, 0.4) | -1.0 | (-2.5, 0.5) |
|  | Other neurological disorders | 0.5*** | (0.4, 0.6) | 0.6 | (-0.3, 1.5) | -0.3 | (-0.6, 0.0) | -0.6* | (-1.1, -0.1) | 0.3 | (-1.0, 1.5) |
|  | Seizures and epilepsy | 0.4** | (0.1, 0.6) | 0.3 | (-1.2, 1.8) | -0.5* | (-1.0, -0.1) | -0.5 | (-1.3, 0.2) | 0.0 | (-1.7, 1.8) |
|  | Obesity | 0.6*** | (0.5, 0.7) | 0.2 | (-0.3, 0.7) | -0.1 | (-0.2, 0.1) | -0.0 | (-0.3, 0.2) | 0.2 | (-0.4, 0.9) |
|  | Paralysis | -1.0*** | (-1.2, -0.7) | 1.1 | (-0.7, 2.9) | 0.3 | (-0.2, 0.9) | -0.5 | (-1.5, 0.5) | 1.4 | (-1.0, 3.9) |
|  | Peripheral vascular disease | 0.3*** | (0.2, 0.4) | 0.3 | (-0.5, 1.1) | -0.3* | (-0.5, -0.0) | 0.5* | (0.0, 0.9) | -0.4 | (-1.4, 0.6) |
|  | Psychoses | 0.1* | (0.0, 0.2) | 0.1 | (-0.5, 0.7) | -0.4*** | (-0.6, -0.2) | -0.1 | (-0.4, 0.3) | -0.3 | (-1.1, 0.6) |
|  | Pulmonary circulation disorder | -0.7*** | (-0.9, -0.5) | 0.6 | (-1.1, 2.3) | 0.1 | (-0.4, 0.5) | -0.5 | (-1.5, 0.4) | 0.3 | (-1.7, 2.3) |
|  | Renal failure, moderate | -0.0 | (-0.2, 0.1) | 0.0 | (-0.8, 0.9) | 0.0 | (-0.2, 0.3) | 0.1 | (-0.4, 0.7) | -0.0 | (-1.2, 1.2) |
|  | Renal failure, severe | -0.3** | (-0.6, -0.1) | -1.2 | (-2.6, 0.2) | -0.5* | (-0.9, -0.0) | -0.3 | (-1.2, 0.5) | 1.1 | (-1.0, 3.1) |
|  | Renal failure, unknown severity | 0.2 | (-0.0, 0.5) | 0.4 | (-1.1, 1.9) | 0.2 | (-0.2, 0.6) | -0.2 | (-1.1, 0.8) | 1.6 | (-0.8, 3.9) |
|  | Hypothyroidism | -0.1* | (-0.2, -0.0) | 0.3 | (-0.5, 1.1) | -0.0 | (-0.3, 0.3) | 0.2 | (-0.2, 0.6) | -0.8 | (-1.8, 0.2) |
|  | Other thyroid disorders | 0.2 | (-0.0, 0.4) | 0.9 | (-0.4, 2.1) | -0.3 | (-0.6, 0.1) | 0.1 | (-0.6, 0.9) | -0.9 | (-2.4, 0.7) |
|  | Peptic ulcer disease | -1.3*** | (-1.6, -1.0) | -0.3 | (-2.4, 1.8) | -0.0 | (-0.7, 0.6) | 0.0 | (-1.1, 1.2) | -0.6 | (-3.0, 1.8) |
|  | Valvular disease | 1.6*** | (1.5, 1.8) | 0.3 | (-0.9, 1.6) | -0.4* | (-0.8, -0.0) | -0.1 | (-0.8, 0.6) | -0.6 | (-2.1, 1.0) |
|  | Weight loss | -3.0*** | (-3.2, -2.8) | 0.5 | (-0.6, 1.6) | -0.0 | (-0.3, 0.3) | 0.2 | (-0.5, 0.9) | -0.1 | (-1.6, 1.3) |
| Year | |  |  |  |  |  |  |  |  |  |  |
|  | 2018 | ref | ref | ref | ref | ref | ref | ref | ref | ref | ref |
|  | 2019 | 0.1* | (0.0, 0.2) | 0.5 | (-0.0, 1.0) | 0.3** | (0.1, 0.5) | 1.0*** | (0.7, 1.4) | -0.3 | (-1.1, 0.5) |
|  | 2020 | 4.1*** | (3.9, 4.2) | -0.4 | (-1.2, 0.3) | -0.1 | (-0.4, 0.1) | 0.6** | (0.1, 1.0) | -0.7 | (-1.7, 0.4) |
|  | 2021 | 3.8*** | (3.7, 3.9) | 1.2*** | (0.6, 1.8) | 0.7*** | (0.5, 0.9) | 2.4*** | (2.1, 2.8) | -0.4 | (-1.3, 0.4) |
|  | 2022 | 6.4*** | (6.3, 6.5) | 2.6*** | (2.0, 3.2) | 1.1*** | (0.9, 1.3) | 3.6*** | (3.2, 4.0) | 0.6 | (-0.3, 1.5) |
| **Notes:** AA/PI = Asian American/Pacific Islander. AI/AN = American Indian/Alaska Native. The table displays regression-adjusted associations between patient characteristics and wait times for new patient referrals to specialty care (in days), stratified by racial group. For brevity, estimates for individual comorbidities, county-level characteristics, referral year and month, and referring facility are not shown. *p<0.05 **p<0.01 ***p<0.001. Differences were assessed using regression interactions with white Veterans as the reference group; see main text for more details. ^1^Veterans are classified into eight priority groups as outlined by the VA. Groups 1 and 4 constitute those with serious service-connected disabilities (greater than 50% disability or housebound); groups 2, 3, and 6 are those with low or moderate service-connected disabilities; group 5 comprises those with economic hardships; and groups 7 and 8 have no service-connected disabilities and household incomes above certain thresholds. Veterans cannot be members of multiple priority groups; low-income veterans with service-connected disabilities are categorized according to their disability rating. | | | | | | | | | | | |

| **Appendix A7. Kitagawa-Oaxaca-Blinder decomposition for racial and ethnic disparities in appointment wait times for Veterans Health Administration specialists** | | | | | |  |
| --- | --- | --- | --- | --- | --- | --- |
|  | |  | **Asian/Pacific Islander** | **Black** | **Hispanic** | **Native American/ Alaska Native** |
| Explained difference^1^ | | | 0.7*** | 2.6*** | 2.6*** | 1.7*** |
| Unexplained difference^2^ | | | 0.3 | 0.3 | -1.3*** | -0.2 |
| Source of difference^3^ | | |  |  |  |  |
|  | | Endowment effects | 0.7*** | 2.0*** | 1.6*** | 1.4*** |
|  | | Coefficient effects | 0.8*** | 1.4*** | 2.3*** | 0.7** |
|  | | Interaction effects | -0.8*** | -0.8*** | -1.3*** | -0.4** |
| Endowment effects | | |  |  |  |  |
|  | | Female | 0.0 | -0.1*** | 0.0 | 0.0 |
|  | | Age | -0.4*** | -0.1*** | -0.6*** | -0.2** |
|  | | Rural | 0.0 | 0.0 | 0.0 | 0.0 |
|  | | Priority group | 0.1 | 0.0 | 0.0* | 0.0 |
|  | | Elixhauser comorbidities | 0.1 | -0.2*** | 0.1* | -0.1 |
|  | | Referral specialty | 0.0 | 0.5*** | 0.3*** | 0.2*** |
|  | | County: Median household income | 0.1 | 0.0 | 0.0 | 0.0 |
|  | | County: % with a college degree | 0.0 | -0.1* | 0.2*** | 0.0 |
|  | | County: Median home value | 0.2 | 0.0 | 0.0 | 0.0 |
|  | | County: # of primary care physicians | -0.1 | 0.1* | 0.0 | 0.0 |
|  | | County: # of specialist physicians | 0.0 | -0.1 | 0.0 | 0.0 |
|  | | Referral month | 0.0** | 0.0 | 0.0 | 0.0 |
|  | | Referral year | 0.3*** | 0.1*** | 0.3*** | 0.0 |
|  | | Referring facility | 0.4** | 1.7*** | 1.3*** | 1.4*** |
| Coefficient effects | | |  |  |  |  |
|  | | Female | 0.0 | -0.1** | -0.1* | 0.0 |
|  | | Age | -0.1 | -0.1** | 0.0 | -0.1 |
|  | | Rural | 0.0 | 0.0 | 0.0 | -0.1 |
|  | | Priority group | 0.1 | 0.0 | 0.1 | 0.0 |
|  | | Elixhauser comorbidities | 0.3 | 0.0 | 0.2 | 0.8* |
|  | | Referral specialty | -0.1 | 0.3*** | 0.4*** | 0.0 |
|  | | County: Median household income | 0.1 | -0.3*** | -0.2 | 0.1 |
|  | | County: % with a college degree | -0.1 | -0.4*** | 0.5** | -0.3 |
|  | | County: Median home value | 0.7 | 0.6*** | 0.4* | -0.1 |
|  | | County: # of primary care physicians | -0.1 | 0.4** | -0.2 | 0.3 |
|  | | County: # of specialist physicians | -0.4 | -0.4* | -0.1 | 0.2 |
|  | | Referral month | 0.0 | 0.0 | 0.0 | 0.0 |
|  | | Referral year | 0.2*** | 0.1*** | 0.2*** | 0.0 |
|  | | Referring facility | 0.3 | 1.4*** | 1.3*** | 0.0 |
| Interaction effects | | |  |  |  |  |
|  | | Female | 0.0 | 0.0 | 0.0 | 0.0 |
|  | | Age | -0.1* | 0.1** | 0.0 | 0.0 |
|  | | Rural | 0.0 | 0.0 | 0.0 | 0.0 |
|  | | Priority group | -0.1 | 0.0 | 0.0 | 0.0 |
|  | | Elixhauser comorbidities | 0.1 | -0.1** | 0.0 | 0.0 |
|  | | Referral specialty | -0.2*** | -0.2*** | -0.4*** | -0.2*** |
|  | | County: Median household income | -0.1 | -0.0*** | 0.0 | 0.1 |
|  | | County: % with a college degree | 0.1 | 0.1*** | -0.1*** | 0.0 |
|  | | County: Median home value | -0.3 | -0.0*** | -0.1 | -0.1 |
|  | | County: # of primary care physicians | 0.0 | -0.1*** | 0.1 | 0.0 |
|  | | County: # of specialist physicians | 0.1 | 0.1** | 0.0 | 0.0 |
|  | | Referral month | 0.0 | 0.0 | 0.0 | 0.0 |
|  | | Referral year | -0.1*** | 0.0 | -0.1*** | 0.0 |
|  | | Referring facility | -0.2 | -0.6*** | -0.7*** | -0.2 |
| **Notes:** The table displays results of the Kitagawa-Oaxaca-Blinder decomposition of differences in appointment wait times by Veterans' self-reported primary race and ethnicity. All Hispanic Veterans were counted as Hispanic, regardless of primary race. White Veterans are used as the reference group in all calculations. ^1^Indicates the overall differences in wait times (in days) that were attributable to endowment, coefficient, or interaction effects. ^2^Indicates the wait time differences that could not be explained and are represented by the model’s constant term. ^3^The “endowment effect” measures the portion of the disparity that is attributable to group-level differences in observed covariates. The “coefficient effect” measures the portion of the disparity that is attributable to differential group-level associations between the outcome (appointment wait times) and covariates. The “interaction effect” measures the portion of the disparity that is attributable to simultaneous/synergistic effects of the differential covariate levels and associations between racial groups. | | | | | | |

| **Appendix A8. Adjusted associations between Veteran characteristics and wait times for appointments with community-based specialists, by racial group** | | | | | | | | | | | |
| --- | --- | --- | --- | --- | --- | --- | --- | --- | --- | --- | --- |
| **Characteristic** | | **White** | | **AA/PI** | | **Black** | | **Hispanic** | | **AI/AN** | |
|  |  | **Estimate** | **95% CI** | **Estimate** | **95% CI** | **Estimate** | **95% CI** | **Estimate** | **95% CI** | **Estimate** | **95% CI** |
| Gender | |  |  |  |  |  |  |  |  |  |  |
|  | Male | ref | ref | ref | ref | ref | ref | ref | ref | ref | ref |
|  | Female | 1.0*** | (0.8, 1.2) | -0.4 | (-1.3, 0.6) | -0.8*** | (-1.2, -0.4) | -0.7* | (-1.2, -0.2) | -1.0 | (-2.2, 0.2) |
| Rurality | |  |  |  |  |  |  |  |  |  |  |
|  | Non-Rural | ref | ref | ref | ref | ref | ref | ref | ref | ref | ref |
|  | Rural | 0.9*** | (0.8, 1.0) | 0.2 | (-0.7, 1.1) | -0.4 | (-0.8, 0.0) | 0.4 | (-0.1, 0.8) | 1.5** | (0.5, 2.4) |
| Age in years | |  |  |  |  |  |  |  |  |  |  |
|  | <40 | ref | ref | ref | ref | ref | ref | ref | ref | ref | ref |
|  | 40-54 | 0.5*** | (0.2, 0.7) | 0.1 | (-0.9, 1.2) | 0.7** | (0.2, 1.3) | 0.1 | (-0.4, 0.6) | -0.1 | (-1.6, 1.3) |
|  | 55-64 | 0.2 | (-0.1, 0.4) | 0.1 | (-1.0, 1.2) | 0.6* | (0.1, 1.2) | 0.5 | (-0.1, 1.0) | -0.3 | (-1.7, 1.2) |
|  | 65-79 | -0.3** | (-0.5, -0.1) | 0.5 | (-0.7, 1.6) | 1.7*** | (1.1, 2.3) | 0.8** | (0.2, 1.3) | 1.3 | (-0.1, 2.8) |
|  | 80+ | -0.7*** | (-1.0, -0.5) | 1.4 | (-0.1, 3.0) | 3.0*** | (2.2, 3.8) | 1.6*** | (0.8, 2.4) | 2.0* | (0.1, 4.0) |
| Priority Group^1^ | |  |  |  |  |  |  |  |  |  |  |
|  | Groups 1, 4 | 0.2** | (0.1, 0.4) | 1.3* | (0.1, 2.5) | -0.2 | (-0.7, 0.3) | 0.0 | (-0.6, 0.6) | 2.1** | (0.7, 3.5) |
|  | Groups 2, 3, 6 | 0.0 | (-0.1, 0.2) | 1.5* | (0.2, 2.8) | 0.3 | (-0.3, 0.8) | 0.4 | (-0.2, 1.1) | 2.1** | (0.6, 3.7) |
|  | Group 5 | 0.1 | (-0.1, 0.3) | 1.9* | (0.4, 3.5) | 0.5 | (-0.1, 1.1) | 0.4 | (-0.3, 1.1) | 1.8* | (0.2, 3.4) |
|  | Groups 7, 8 | ref | ref | ref | ref | ref | ref | ref | ref | ref | ref |
| Specialty | |  |  |  |  |  |  |  |  |  |  |
|  | Cardiology | -7.0*** | (-7.2, -6.7) | -5.6*** | (-7.6, -3.6) | -3.3*** | (-4.0, -2.5) | -2.8*** | (-3.6, -1.9) | -0.6 | (-2.7, 1.5) |
|  | Dermatology | 5.7*** | (5.5, 5.9) | -11.5*** | (-13.4, -9.6) | -0.7 | (-1.6, 0.1) | -3.9*** | (-4.8, -2.9) | 3.4** | (1.2, 5.7) |
|  | Otorhinolaryngology | -3.1*** | (-3.4, -2.8) | -6.0*** | (-8.1, -3.9) | -3.6*** | (-4.5, -2.7) | -2.2*** | (-3.2, -1.2) | 3.1* | (0.6, 5.6) |
|  | Gastroenterology | 11.6*** | (11.4, 11.8) | -12.2*** | (-14.0, -10.4) | -2.3*** | (-2.9, -1.6) | -4.5*** | (-5.3, -3.7) | 1.0 | (-1.0, 3.0) |
|  | Mental health | -8.6*** | (-8.9, -8.2) | -10.0*** | (-12.0, -8.0) | -6.1*** | (-7.0, -5.2) | -2.2*** | (-3.2, -1.2) | 3.1* | (0.5, 5.8) |
|  | Ophthalmology | -0.5*** | (-0.7, -0.3) | -11.3*** | (-13.0, -9.7) | 0.9** | (0.3, 1.5) | -6.1*** | (-6.8, -5.4) | -0.7 | (-2.4, 1.1) |
|  | Orthopedics | -10.0*** | (-10.3, -9.8) | -9.3*** | (-11.1, -7.5) | 0.7* | (0.0, 1.4) | 0.8* | (0.1, 1.6) | 2.3* | (0.4, 4.2) |
|  | Physical therapy | -5.0*** | (-5.3, -4.7) | -11.6*** | (-13.4, -9.7) | 1.4*** | (0.6, 2.1) | -4.2*** | (-5.0, -3.4) | -0.2 | (-2.3, 1.9) |
|  | Podiatry | -17.2*** | (-17.4, -17.0) | -9.7*** | (-11.3, -8.1) | -2.5*** | (-3.1, -1.9) | -1.7*** | (-2.4, -1.0) | 0.6 | (-1.2, 2.4) |
|  | Urology | ref | ref | ref | ref | ref | ref | ref | ref | ref | ref |
| Comorbidities | |  |  |  |  |  |  |  |  |  |  |
|  | AIDS/HIV | 0.1 | (-0.8, 0.9) | 2.5 | (-3.9, 9.0) | 0.1 | (-1.4, 1.6) | -1.2 | (-3.2, 0.8) | -2.8 | (-8.5, 2.9) |
|  | Alcohol abuse | -0.3*** | (-0.5, -0.1) | -0.7 | (-1.8, 0.4) | -0.2 | (-0.7, 0.2) | 0.4 | (-0.1, 0.8) | 0.3 | (-0.9, 1.4) |
|  | Anemia deficiency | -0.5*** | (-0.6, -0.4) | 0.5 | (-0.6, 1.6) | 0.2 | (-0.1, 0.6) | 0.0 | (-0.5, 0.5) | 1.2 | (-0.0, 2.4) |
|  | Autoimmune conditions | -0.1 | (-0.4, 0.1) | 1.1 | (-0.4, 2.5) | 0.5 | (-0.1, 1.1) | -0.2 | (-0.9, 0.5) | 0.6 | (-1.1, 2.2) |
|  | Blood loss anemia | -1.1*** | (-1.6, -0.6) | -3.9* | (-7.1, -0.8) | 0.1 | (-1.1, 1.3) | 1.6* | (0.1, 3.1) | -0.4 | (-4.2, 3.3) |
|  | Leukemia | -0.6* | (-1.2, -0.1) | 1.9 | (-3.0, 6.7) | -1.3 | (-3.4, 0.7) | 0.3 | (-2.1, 2.8) | -3.7 | (-7.7, 0.2) |
|  | Lymphoma | -1.5*** | (-2.0, -1.0) | 0.9 | (-2.5, 4.4) | -0.5 | (-1.8, 0.9) | -0.2 | (-2.1, 1.6) | -2.9 | (-7.5, 1.7) |
|  | Metastatic Cancer | -3.2*** | (-3.7, -2.8) | 0.0 | (-3.8, 3.8) | -0.0 | (-1.4, 1.3) | 1.7 | (-0.3, 3.6) | -2.1 | (-5.8, 1.6) |
|  | Solid tumor without metastasis, in situ | -0.5*** | (-0.7, -0.3) | -1.6 | (-3.2, 0.0) | 0.3 | (-0.3, 1.0) | 0.2 | (-0.7, 1.2) | -0.3 | (-2.2, 1.6) |
|  | Solid tumor without metastasis, malignant | -1.5*** | (-1.6, -1.3) | -0.6 | (-1.9, 0.7) | 0.4 | (-0.1, 0.8) | 0.5 | (-0.2, 1.1) | -0.2 | (-1.8, 1.4) |
|  | Cerebrovascular disease, present on admission | -0.3*** | (-0.5, -0.2) | -0.1 | (-1.0, 0.7) | -0.4 | (-0.7, 0.0) | -0.2 | (-0.7, 0.2) | 0.3 | (-0.7, 1.3) |
|  | Cerebrovascular disease, sequela | 0.5* | (0.1, 0.8) | 0.6 | (-2.3, 3.5) | -0.6 | (-1.7, 0.5) | -0.3 | (-1.7, 1.0) | 0.5 | (-2.6, 3.5) |
|  | Coagulopathy | -0.6*** | (-0.9, -0.4) | 0.4 | (-1.8, 2.5) | -0.5 | (-1.4, 0.3) | 0.6 | (-0.2, 1.5) | -1.9 | (-4.2, 0.4) |
|  | Dementia | 1.2*** | (1.0, 1.5) | 2.4* | (0.2, 4.6) | -0.2 | (-1.2, 0.7) | 0.2 | (-0.8, 1.2) | -2.1 | (-4.4, 0.2) |
|  | Depression | 0.4*** | (0.3, 0.5) | -0.1 | (-0.8, 0.5) | -0.2 | (-0.5, 0.0) | -0.0 | (-0.3, 0.3) | 0.3 | (-0.5, 1.1) |
|  | Diabetes with chronic complications | 0.2** | (0.1, 0.3) | 0.7 | (-0.2, 1.6) | -0.1 | (-0.5, 0.3) | -0.1 | (-0.5, 0.4) | -0.1 | (-1.2, 1.0) |
|  | Diabetes without chronic complications | 0.4*** | (0.3, 0.6) | -0.4 | (-1.3, 0.4) | -0.2 | (-0.6, 0.1) | 0.1 | (-0.3, 0.5) | 0.5 | (-0.5, 1.5) |
|  | Drug abuse | -0.2 | (-0.4, 0.0) | 0.3 | (-1.2, 1.8) | -0.6* | (-1.1, -0.1) | -0.4 | (-1.0, 0.2) | -0.3 | (-1.7, 1.2) |
|  | Fluid and electrolyte disorders | -0.9*** | (-1.1, -0.7) | -0.1 | (-1.4, 1.2) | 0.4 | (-0.1, 0.8) | 0.1 | (-0.5, 0.7) | -0.3 | (-1.7, 1.1) |
|  | Heart Failure | 0.4*** | (0.3, 0.6) | 0.0 | (-1.4, 1.4) | 0.0 | (-0.5, 0.6) | -0.1 | (-0.9, 0.6) | 0.2 | (-1.4, 1.7) |
|  | Homelessness | -0.9*** | (-1.2, -0.6) | -0.7 | (-2.6, 1.3) | 0.1 | (-0.5, 0.8) | 0.3 | (-0.6, 1.1) | 1.0 | (-0.9, 2.8) |
|  | Hypertension with chronic complications | 0.0 | (-0.2, 0.2) | 0.8 | (-0.4, 2.0) | -0.4 | (-0.8, 0.1) | -0.3 | (-0.9, 0.3) | -0.2 | (-1.6, 1.3) |
|  | Hypertension without chronic complications | 0.1 | (-0.0, 0.2) | 0.4 | (-0.4, 1.1) | -0.3 | (-0.6, 0.0) | 0.2 | (-0.1, 0.6) | -0.2 | (-1.1, 0.6) |
|  | Liver disease, mild | -0.4*** | (-0.6, -0.2) | 1.1 | (-0.0, 2.2) | 0.3 | (-0.2, 0.8) | -0.0 | (-0.5, 0.5) | -0.4 | (-1.7, 1.0) |
|  | Liver disease, severe | -0.2 | (-0.8, 0.3) | 1.5 | (-2.9, 5.9) | 1.8 | (-0.1, 3.7) | -1.0 | (-2.6, 0.6) | 5.4* | (1.2, 9.6) |
|  | Liver disease, unknown severity | -2.5** | (-4.2, -0.8) | 3.5 | (-11.0, 18.1) | -0.9 | (-5.9, 4.0) | 3.6 | (-1.4, 8.6) | 5.3 | (-7.7, 18.2) |
|  | Chronic pulmonary disease | 0.2** | (0.1, 0.3) | 0.5 | (-0.4, 1.3) | -0.1 | (-0.4, 0.3) | -0.2 | (-0.6, 0.3) | -0.1 | (-1.0, 0.8) |
|  | Neurological disorders affecting movement | 0.0 | (-0.2, 0.3) | -1.3 | (-3.1, 0.5) | 0.2 | (-0.7, 1.2) | -0.5 | (-1.4, 0.3) | -2.0* | (-3.9, -0.0) |
|  | Other neurological disorders | 0.3** | (0.1, 0.5) | 0.4 | (-1.1, 1.8) | -0.5 | (-1.1, 0.2) | -0.1 | (-0.8, 0.7) | -0.4 | (-2.1, 1.4) |
|  | Seizures and epilepsy | 0.1 | (-0.2, 0.5) | -0.7 | (-3.2, 1.8) | 0.4 | (-0.5, 1.2) | 0.7 | (-0.4, 1.7) | 0.6 | (-1.6, 2.7) |
|  | Obesity | 0.2*** | (0.1, 0.3) | 0.4 | (-0.3, 1.1) | -0.1 | (-0.3, 0.2) | 0.2 | (-0.1, 0.5) | 0.7 | (-0.1, 1.5) |
|  | Paralysis | -0.1 | (-0.5, 0.3) | -0.5 | (-3.8, 2.8) | -0.3 | (-1.5, 0.8) | 0.1 | (-1.4, 1.5) | -2.2 | (-5.3, 0.9) |
|  | Peripheral vascular disease | -0.2*** | (-0.4, -0.1) | -0.7 | (-1.8, 0.4) | 0.3 | (-0.1, 0.8) | 0.2 | (-0.3, 0.8) | 0.2 | (-1.1, 1.4) |
|  | Psychoses | 0.2* | (0.0, 0.3) | -0.9 | (-1.9, 0.1) | -0.2 | (-0.7, 0.2) | -0.2 | (-0.7, 0.3) | 0.3 | (-0.9, 1.4) |
|  | Pulmonary circulation disorder | -0.5** | (-0.8, -0.2) | -0.1 | (-2.5, 2.3) | -0.1 | (-0.9, 0.8) | 1.4* | (0.1, 2.8) | -2.0 | (-4.6, 0.6) |
|  | Renal failure, moderate | -0.0 | (-0.2, 0.1) | -0.6 | (-1.8, 0.6) | -0.3 | (-0.8, 0.2) | 0.1 | (-0.5, 0.7) | -0.3 | (-1.9, 1.4) |
|  | Renal failure, severe | -0.2 | (-0.5, 0.2) | -1.0 | (-3.1, 1.1) | -0.1 | (-1.0, 0.8) | 1.3* | (0.2, 2.4) | -0.8 | (-3.5, 1.9) |
|  | Renal failure, unknown severity | 0.0 | (-0.3, 0.3) | 1.1 | (-1.1, 3.2) | -0.7 | (-1.5, 0.1) | 0.9 | (-0.3, 2.0) | 3.3* | (0.1, 6.4) |
|  | Hypothyroidism | 0.1* | (0.0, 0.3) | -1.5* | (-2.6, -0.3) | -0.3 | (-0.8, 0.3) | -0.3 | (-0.8, 0.2) | 0.5 | (-0.7, 1.7) |
|  | Other thyroid disorders | -0.2 | (-0.5, 0.1) | 1.0 | (-0.8, 2.8) | -0.4 | (-1.1, 0.3) | 0.1 | (-1.0, 1.1) | 1.5 | (-1.0, 4.0) |
|  | Peptic ulcer disease | -1.1*** | (-1.5, -0.6) | 3.5* | (0.1, 6.9) | -0.1 | (-1.3, 1.2) | -1.3 | (-2.8, 0.3) | -1.0 | (-4.6, 2.5) |
|  | Valvular disease | -0.2 | (-0.4, 0.0) | -0.1 | (-1.6, 1.5) | 0.1 | (-0.6, 0.9) | -0.1 | (-1.0, 0.7) | 0.3 | (-1.5, 2.2) |
|  | Weight loss | -1.1*** | (-1.4, -0.9) | 0.2 | (-1.6, 2.0) | 0.1 | (-0.6, 0.7) | 0.5 | (-0.4, 1.5) | 0.9 | (-1.2, 3.0) |
| Year | |  |  |  |  |  |  |  |  |  |  |
|  | 2018 | ref | ref | ref | ref | ref | ref | ref | ref | ref | ref |
|  | 2019 | 4.8*** | (4.7, 5.0) | -0.5 | (-1.4, 0.3) | 1.1*** | (0.7, 1.5) | 1.3*** | (0.9, 1.7) | -1.2* | (-2.3, -0.2) |
|  | 2020 | 8.8*** | (8.7, 9.0) | -2.3*** | (-3.4, -1.3) | -3.0*** | (-3.5, -2.6) | -2.3*** | (-2.8, -1.7) | -1.2 | (-2.4, 0.0) |
|  | 2021 | 9.1*** | (8.9, 9.3) | 5.8*** | (4.8, 6.9) | 4.6*** | (4.2, 5.1) | 1.2*** | (0.7, 1.7) | 0.5 | (-0.7, 1.7) |
|  | 2022 | 9.8*** | (9.6, 9.9) | -0.1 | (-1.0, 0.9) | 1.0*** | (0.6, 1.4) | 1.5*** | (1.0, 1.9) | -0.8 | (-1.9, 0.4) |
| **Notes:** AA/PI = Asian American/Pacific Islander. AI/AN = American Indian/Alaska Native. The table displays regression-adjusted associations between patient characteristics and wait times for new patient referrals to specialty care (in days), stratified by racial group. For brevity, estimates for individual comorbidities, county-level characteristics, referral year and month, and referring facility are not shown. *p<0.05 **p<0.01 ***p<0.001. Differences were assessed using regression interactions with white Veterans as the reference group; see main text for more details. ^1^Veterans are classified into eight priority groups as outlined by the VA. Groups 1 and 4 constitute those with serious service-connected disabilities (greater than 50% disability or housebound); groups 2, 3, and 6 are those with low or moderate service-connected disabilities; group 5 comprises those with economic hardships; and groups 7 and 8 have no service-connected disabilities and household incomes above certain thresholds. Veterans cannot be members of multiple priority groups; low-income veterans with service-connected disabilities are categorized according to their disability rating. | | | | | | | | | | | |

| **Appendix A9. Kitagawa-Oaxaca-Blinder decomposition for racial and ethnic disparities in appointment wait times for community-based specialists** | | | | | |
| --- | --- | --- | --- | --- | --- |
|  |  | **Asian/Pacific Islander** | **Black** | **Hispanic** | **Native American/ Alaska Native** |
| Explained difference^1^ | | -0.3*** | 4.8*** | -4.4*** | 2.1*** |
| Unexplained difference^2^ | | 1.7** | 2.9*** | 1.3*** | -0.7 |
| Source of difference^3^ | |  |  |  |  |
|  | Endowment effects | 0.3 | 5.2*** | -4.3*** | 0.4* |
|  | Coefficient effects | -0.4*** | -0.1*** | -0.5*** | 1.6*** |
|  | Interaction effects | -0.2 | -0.3*** | 0.4** | 0.1 |
| Endowment effects | |  |  |  |  |
|  | Female | 0.0 | 0.0 | 0.0 | 0.0 |
|  | Age | 0.0 | -0.2*** | -0.1 | -0.1 |
|  | Rural | -0.3* | -0.2** | -0.4*** | 0.1*** |
|  | Priority group | 0.0 | 0.0 | 0.0 | 0.1* |
|  | Elixhauser comorbidities | 0.2** | 0.0 | 0.2*** | 0.2** |
|  | Referral specialty | -1.3*** | -0.4*** | -0.8*** | -0.9*** |
|  | County: Median household income | -0.6* | 0.0 | 0.0 | 0.4*** |
|  | County: % with a college degree | -0.1 | 0.0 | -0.1*** | 0.0 |
|  | County: Median home value | 0.2 | 0.0 | -0.3*** | -0.1 |
|  | County: # of primary care physicians | 0.2 | -0.1** | -0.1* | 0.0 |
|  | County: # of specialist physicians | -0.4* | 0.2** | -0.2** | 0.0 |
|  | Referral month | 0.0 | 0.0 | 0.0 | 0.0 |
|  | Referral year | 0.0 | -0.1*** | 0.0 | -0.1*** |
|  | Referring facility | 2.2*** | 6.0*** | -2.5*** | 0.8*** |
| Coefficient effects | |  |  |  |  |
|  | Female | -0.1 | -0.2*** | -0.1* | -0.1 |
|  | Age | -0.1 | -0.1* | -0.1* | 0.1 |
|  | Rural | 0.1 | -0.1 | 0.1 | 0.8** |
|  | Priority group | 0.1 | -0.2* | -0.1 | 0.4* |
|  | Elixhauser comorbidities | 0.2 | -0.6*** | 0.2 | 0.5 |
|  | Referral specialty | -1.0*** | 0.7*** | -0.7*** | -0.6*** |
|  | County: Median household income | -0.4 | 0.1* | -0.2** | -0.1 |
|  | County: % with a college degree | 0.1 | 0.1 | -0.3** | 0.3 |
|  | County: Median home value | 0.5 | -0.7*** | 0.2 | 0.2 |
|  | County: # of primary care physicians | 0.5 | -0.3* | 0.3* | -0.1 |
|  | County: # of specialist physicians | -0.3 | 0.9*** | -0.2 | 0.0 |
|  | Referral month | 0.0 | 0.0 | 0.0 | 0.0 |
|  | Referral year | 0.1** | 0.1*** | 0.1*** | 0.0 |
|  | Referring facility | -0.1 | 0.1 | 0.2 | 0.1 |
| Interaction effects | |  |  |  |  |
|  | Female | 0.0 | 0.1*** | 0.0 | 0.1 |
|  | Age | 0.1 | 0.4*** | 0.2*** | 0.2** |
|  | Rural | 0.0 | -0.1 | 0.1 | 0.0 |
|  | Priority group | 0.0 | 0.1** | 0.0 | -0.1* |
|  | Elixhauser comorbidities | -0.1 | 0.1 | 0.0 | -0.2* |
|  | Referral specialty | 0.1 | 0.0 | 0.0 | 0.1 |
|  | County: Median household income | 0.2 | 0.1* | 0.0 | -0.3** |
|  | County: % with a college degree | -0.1 | 0.0 | 0.1** | 0.1 |
|  | County: Median home value | -0.2 | 0.0 | 0.3*** | 0.1 |
|  | County: # of primary care physicians | -0.2 | 0.1*** | 0.0 | 0.0 |
|  | County: # of specialist physicians | 0.2 | -0.4*** | 0.1 | 0.0 |
|  | Referral month | 0.0 | 0.0 | 0.0 | 0.0 |
|  | Referral year | 0.0 | 0.0 | 0.0 | 0.0 |
|  | Referring facility | -0.2 | -0.6*** | -0.4* | 0.1 |
| **Notes:** The table displays results of the Kitagawa-Oaxaca-Blinder decomposition of differences in appointment wait times by Veterans' self-reported primary race and ethnicity. All Hispanic Veterans were counted as Hispanic, regardless of primary race. White Veterans are used as the reference group in all calculations. ^1^Indicates the overall differences in wait times (in days) that were attributable to endowment, coefficient, or interaction effects. ^2^Indicates the wait time differences that could not be explained and are represented by the model’s constant term. ^3^The “endowment effect” measures the portion of the disparity that is attributable to group-level differences in observed covariates. The “coefficient effect” measures the portion of the disparity that is attributable to differential group-level associations between the outcome (appointment wait times) and covariates. The “interaction effect” measures the portion of the disparity that is attributable to simultaneous/synergistic effects of the differential covariate levels and associations between racial groups. | | | | | |
